# Humid periods over western Sahara during the Middle Miocene Climate Transition and their implications on the carbon cycle

**DOI:** 10.1126/sciadv.aec7963

**Published:** 2026-11-06

**Authors:** Ray Zammit, Caroline H. Lear, Lucas J. Lourens, Paul N. Pearson, Amy Thomas-Sparkes, Marcus P. S. Badger

**Affiliations:** ^1^ School of Earth and Environmental Sciences, Cardiff University, UK.; ^2^ Department of Mathematics and Science Education, University of Malta, Malta.; ^3^ Faculty of Geosciences, Utrecht University, The Netherlands.; ^4^ School of Environment, Earth and Ecosystem Sciences, The Open University, UK.

## Abstract

The Middle Miocene expansion of the Antarctic Ice Sheet coincides with major sedimentological changes in Malta. This interval also saw reorganisations in ocean circulation linked to closure of the Mesopotamian Seaway (MSR-events) and perturbations in the carbon cycle (CM events). The final CM event (CM-6), slightly lags the main glacial phase and remains difficult to interpret due to competing mechanisms and conflicting *p*CO_2_ reconstructions. To explore links between cryosphere, circulation, hydroclimate, and the carbon cycle, we present new geochemical records from Malta. Our records show that cryospheric expansion was followed by large, precessionally paced variations in sea-surface salinity and a marked regional increase in biological productivity. The results indicate a shift toward stronger monsoonal influence, delivering enhanced freshwater and nutrient fluxes to the region. This enhanced local biological carbon pump may have acted as a positive feedback to a global CO_2_ drawdown. However, any contribution to global CO_2_ drawdown remains tentative and cannot be confirmed without contemporaneous high-resolution open-ocean records.

## INTRODUCTION

Global continental configurations from the mid-Cretaceous (∼100 Ma) favored a low-latitude circum-global circulation system until oceanic gateways in Indonesia, Mesopotamia and Central America sequentially closed during the Cenozoic (*1*). This system utilized the vast Tethys Ocean as a global corridor for heat and salt transport. Such a system has not been possible since the Middle Miocene Climate Transition (MMCT) due to the shrinkage of the Tethys basin, closure of Mesopotamian Seaway and the formation of a semi-enclosed Mediterranean Sea during the Miocene (*2*) (Fig. 1, A and B). The MMCT was a step-wise transition into a colder global climatic regime ∼14.7 Ma to ∼12.8 Ma (*3*) including expansion of the East Antarctic Ice Sheet, an associated sea-level fall, major perturbations to the global carbon cycle (CM events) recorded in the global benthic foraminiferal carbon isotope record (*4*), and a decline in atmospheric *p*CO_2_ levels (*5*). It is therefore instructive to ask how this major change in oceanic circulation may have affected local and global hydrology, climate, and carbon cycle dynamics in order to understand Earth system processes.

**Fig. 1.**
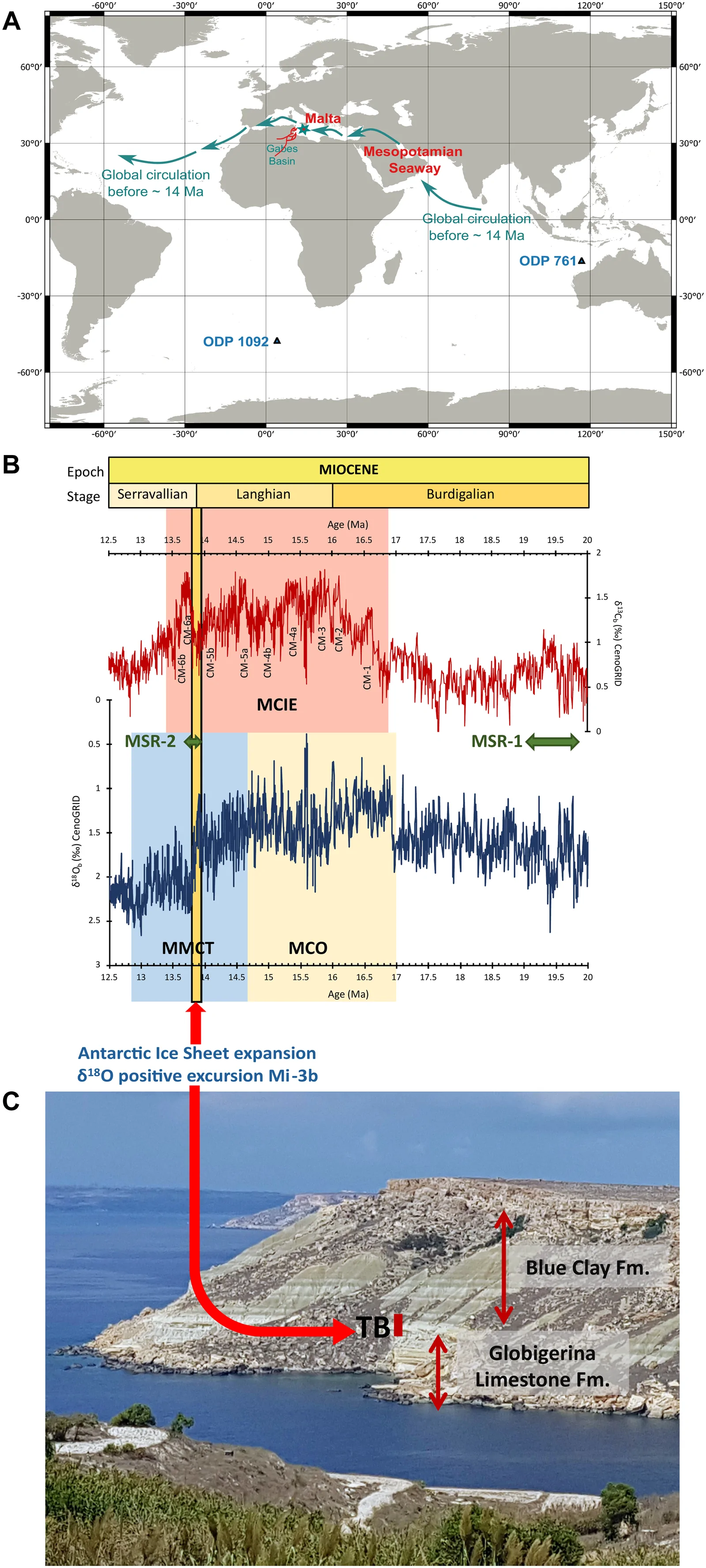
Middle Miocene geography, climate and the records from Malta. (**A**) Prior to the Middle Miocene Climate Transition (∼14 Ma) surface ocean waters could flow from the Indian to the Atlantic Ocean via the Mesopotamian Seaway and the Mediterranean (*2*). (**B**) Global benthic δ^18^O and δ^13^C records indicate a highly dynamic climate system (*4*). The Miocene Climatic Optimum (MCO), Monterey Carbon Isotope Excursion (MCIE) and carbon maxima (CM) events are shown. The CM-6 event occurs during the Middle Miocene Climate Transition (MMCT). MSR-1 and MSR-2 (Mesopotamian Seaway Restriction events) represent Early and Middle Miocene events of restriction of water flow through the Mesopotamian Seaway respectively (*2*). Foraminifera from the Ras il-Pellegrin section in Malta (**C**) hold a geochemical record of the MMCT. The shift from the marine Globigerina Limestone formation to the terrestrially influenced Blue Clay via a transition bed (TB) occurs during the MMCT and hosts the Langhian-Serravallian GSSP (Global Stratotype Section and Point).

Records from Miocene sedimentary successions now uplifted and exposed on the Maltese Islands have been used to provide information about regional and global climate and oceanography during the Miocene (*2*, *6*–*16*). This has included studies related to the global carbon cycle (*7*, *16*), North African hydroclimate variability (*6*, *12*, *13*), global stratigraphy (*9*, *10*, *17*) and oceanic gateway changes (*2*, *11*) (Fig. 1A). The εNd records from Malta and the Maldives show that the Mesopotamian Seaway closed in two distinct Mesopotamian Seaway Restriction (MSR) events during the Miocene (*2*). The first of these (MSR-1) occurred during the Early Miocene (20.35–19.05 Ma) (*2*, *13*), while the second and terminal disconnection (MSR-2) occurred during the MMCT (13.83–13.66 Ma) (*2*, *13*) (Fig. 1B). Following MSR-1, a change from marine carbonates to clay-rich sediments, increased sedimentation rates, and multiproxy geochemical records from the il-Blata section in southwest Malta indicate a transition from an arid to a humid climate (*13*). Furthermore, the identification of the earliest recorded Mediterranean sapropelic layers (∼1.4% total organic carbon) in association with iron-dolomite at il-Blata has been interpreted as indicating an influx of fresh water, leading to surface fertilisation and water column stratification in the area (*12*). The most likely source of this discharge of fresh water, clastic material and nutrients appears to have been now extinct river systems (*18*) with deltas in the Gulf of Gabes (Tunisia) (*12*). These observations are consistent with the establishment of a short term monsoonal-like climate over western Sahara and a perturbation in the general circulation at low latitudes related to the initial restriction of the Mesopotamian Seaway (*12*, *13*).

The second event (MSR-2) (*2*) has been correlated to the largest positive oxygen isotope (δ^18^O) excursion (Mi-3b) of the MMCT, associated with a period of Antarctic Ice Sheet growth ending at 13.82 Ma (Fig. 1) (*3*, *5*, *9*, *17*). It is also correlatable with the transition bed (TB) between the Globigerina Limestone Formation and the Blue Clay Formation in the Ras il-Pellegrin (RIP) section in Malta (Fig. 1C). In addition to representing a marked change in local lithology, this transition bed also contains the Global Stratotype Section and Point (GSSP) for the base of the Serravallian (Fig. 1, B and C) (*17*). It is possible that this marked change in sediment lithology reflects a shift in North African hydroclimate similar to MSR-1 (*13*), although the region was also subject to active tectonics at this time, so local changes in relative sea level may also have had an influence (*11*, *19*). It has previously been noted that the planktic foraminifera (surface water) oxygen isotope (δ^18^O) records from the Middle Miocene Marsalforn and Ras il-Pellegrin composite sections in Malta exhibit high amplitude variability (1 ‰ to 2 ‰) following Mi-3b (*9*). It has been suggested that this could be due to high and variable freshwater fluxes in the region (*9*) therefore suggesting a possible increase in hydroclimatic activity in the region. However, this is yet to be tested as the δ^18^O record incorporates signals of temperature, ice volume, and salinity.

While it is likely that the eustatic sea level fall at Mi-3b contributed to MSR-2, the consequent regional and global impacts of the seaway closure are largely unexplored. Such a large change in the long-standing global oceanic circulation coupled with rapid Antarctic glaciation likely affected Mediterranean hydrology and this in turn may have influenced the regional and possibly global carbon cycle. Although previous studies from the il-Blata site in Malta (*12*, *13*) have addressed links between gateway closure and Central Mediterranean hydroclimate, it has not been possible to investigate these relationships at the necessary high-resolution using the il-Blata records.

In addition, while the overall MMCT was associated with decreasing CO_2_ (*5*), the carbon cycle feedbacks at the time are unclear. It has been suggested that the expansion of the East Antarctic Ice Sheet associated with the Mi-3b excursion would have blanketed vast amounts of land area, reducing chemical weathering, and resulting in an increase in atmospheric *p*CO_2_ (*20*, *21*). This is similar to the mechanism proposed during the Eocene-Oligocene Transition Antarctic Ice Sheet expansion (*22*). However, foraminiferal δ^11^B records from the RIP section in Malta (*16*) have been used to suggest that atmospheric *p*CO_2_ declined following the Mi-3b δ^18^O excursion, indicating that CM-6 was associated with decreasing, and not increasing, *p*CO_2_. This led to the proposal that CM-6 actually represented a positive feedback in the climate system, with enhanced organic carbon burial linked with steep polar to low latitude gradients associated with the expansion of the Antarctic Icesheet (*16*). However, enhanced export productivity has not been clearly linked with the timing or amplitude of the δ^13^C positive excursions at high productivity regions like DSDP Site 588 (South Pacific), ODP Site 1171 (South Tasman Rise), or ODP Site 747 (Kerguelen Plateau) (*23*). This suggests that these CM events do not necessarily represent a global palaeoproductivity record and that enhanced palaeoproductivity during these events may have been localized in some regions more than others. Therefore it remains unclear whether the individual CM peaks represent global or regional productivity signals (Fig. 1B) (*23*).

Apart for the record from Malta, there exists a more recent high resolution *p*CO_2_ record across the MMCT. This record from the Southern Ocean (ODP Site 1092) (*24*) indicates the opposite of the Malta records and shows an increase in *p*CO_2_ following the glaciation (*16*, *24*, *25*).This Southern Ocean record has been used to propose that the transient *p*CO_2_ increase reconstructed at ODP Site 1092 reflects a global signal, caused by enhanced ocean ventilation transferring CO_2_ from the deep ocean reservoir into the atmosphere. This mechanism would represent a negative feedback associated with the expansion of the Antarctic Ice Sheet, and furthermore implies that the RIP Malta *p*CO_2_ record is dominated by a strong local/regional signal. This apparent mismatch in the *p*CO_2_ during the MMCT and the closely associated CM-6 maximum event demonstrates that the orbital scale carbon cycle dynamics during this rapid climatic step are poorly constrained and that a comparison between them merits further investigation.

This present study aims to assess how the inferred global scale changes in oceanic circulation associated with MSR-2 and contemporaneous with rapid Antarctic glaciation (Mi-3b excursion) affected Mediterranean hydrology and how this in turn may have been related to millennial scale global carbon cycle dynamics. Although previous studies from Malta have addressed relations between gateway closure and Central Mediterranean hydroclimate, the novelty of this present study lies in the ability to assess these interactions at high resolution and relate this to the carbon cycle. This is made possible due to the availability of high-resolution (one sample per ∼9kyr) samples from the Marsalforn and Ras il-Pellegrin sections containing well-preserved foraminifera that allow for detailed quantitative geochemical investigation into these paleoenvironments. The regional and global connections between the MMCT, Mi-3b, CM-6, MSR-2, Middle Miocene humidification of western Sahara, and orbital-scale carbon cycle feedbacks, are here investigated using a combination of previously available stable isotope (*9*) and trace metal (*16*) records from the Marsalforn and Ras il-Pellegrin sections and combined with new high-resolution benthic and planktic foraminiferal stable isotope, and multi-proxy trace metal records from the same sites. Our records span from 14.4 Ma to 12.8 Ma (that is, most of the MMCT interval as conventionally defined) and have been aligned to the new CenoGRID (Cenozoic Global Reference benthic foraminifer carbon and oxygen Isotope Dataset) (*4*) age model. We also reassessed the available age model for Southern Ocean ODP Site 1092 (*24*) and aligned this to CenoGRID to allow for correct comparison with the RIP records (refer to figs. S4 and S5). Foraminiferal Mg/Ca is used to determine sea surface temperature (SST) and bottom water temperature (BWT) records, to allow unambiguous interpretation of the δ^18^O records and thus identify periods of enhanced hydroclimatic activity. Our results indicate substantial activity of western North Africa drainage systems into the Central Mediterranean immediately following the expansion of the East Antarctic Ice Sheet and the closure of the Mesopotamian Seaway. These active drainage systems would have been important conduits for nutrient delivery into the Mediterranean Sea and therefore may have impacted carbon cycling in the region. We investigated their impacts using Δδ^13^C_p-b_ (planktic-benthic foraminiferal δ^13^C gradient), planktic foraminiferal B/Ca_p_, and planktic foraminiferal Mn/Ca_p_ records, which reflect changes in export productivity, surface carbonate chemistry, and bottom water oxygenation respectively. We compare these records with records from other sites to argue for a significant regional increase in export productivity and carbon in the Mediterranean region during the MMCT. We also make use of our new age models to resolve the apparent mismatch between the published *p*CO_2_ records from Malta and the Southern Ocean.

## RESULTS

### Central Mediterranean temperature and salinity variability

Our new δ^18^O records compare very well with the previously produced records from the same site (*9*) (Fig. 2B). The planktic *Trilobatus trilobus* δ^18^O_p_ records display a sharp negative spike around 13.8 Ma followed by high amplitude (∼2 ‰ maximum) and high frequency (∼20kyr) variability (Fig. 2B). There is no significant change in mean *Trilobatus trilobus* δ^18^O_p_ following the Mi-3b excursion (pre Mi-3b ⟨δ^18^O_p_⟩ = −1.59 ‰, post Mi-3b ⟨δ^18^O_p_⟩ = −1.53 ‰). We note that variance (Anova single factor) in the δ^18^O_p_ records for the same transition show a marked difference, changing from 0.038 to 0.074 following Mi-3b (table S3). Unlike the Maltese δ^18^O planktic record, but similar to the global δ^18^O benthic record, the Maltese benthic *Heterolepa dutemplei* δ^18^O_b_ record displays an overall ∼1.4 ‰ increase through the MMCT (Fig. 2, A and B). The Maltese benthic δ^18^O record also lacks the high frequency variability that is observed in the Maltese planktic δ^18^O records (*9*) (Fig. 3B) and demonstrates a significant change in mean value (pre Mi-3b ⟨δ^18^O_b_⟩ = −0.388 ‰, post Mi-3b ⟨δ^18^O_b_⟩ = 0.231 ‰). The variance in the δ^18^O_b_ records changes from 0.044 to a lower value of 0.020 (table S3). The variability in the planktic δ^18^O after 13.8 Ma could be due to changes in local salinity (evaporation/precipitation/runoff), global ice volume, or changes in sea-surface temperature. Our new planktic and benthic Mg/Ca-temperature records (Fig. 3A) were used to disentangle these signals in the δ^18^O records (Methods). We combine the Mg/Ca-SST (Fig. 3B) and planktic δ^18^O to determine the δ^18^O of surface seawater (δ^18^O_swp_), which reflects global ice-volume and local salinity (Fig. 3C). Mg/Ca derived BWT and benthic δ^18^O are used to produce a bottom water δ^18^O_swb_ record, which is dominated by the global ice volume signal. Since we analysed planktic and benthic δ^18^O from the same samples, we calculate both planktic-benthic Δδ^18^O_p-b_ (Fig. 2C) and planktic-benthic seawater gradient Δδ^18^O_swp-swb_ (Fig. 3C) to determine the precipitation/runoff-evaporation signal in the surface δ^18^O_sw_ and thus act as a semi-quantitative proxy for sea surface salinity (Fig. 3C). SST and BWT were only calculated for the Ras il-Pellegrin section (∼13.72 to 12.86 Ma) since the Mg/Ca records from the Marsalforn section display moderately high Mg/Ca values (∼5 to 11 mmol/mol) and elevated Fe/Ca values suggest a diagenetic coating hindering their use for temperature calculations (fig. S9). The calculated SST and BWT records both display a rapid cooling between 13.84 Ma and 13.50 Ma. Both records are then rather stable until 13.28 Ma, following which they demonstrate gentle warming. The variability in the SST record is not large enough to explain the variability in the δ^18^O_p_ record, pointing to a dominant salinity signal.

**Fig. 2.**
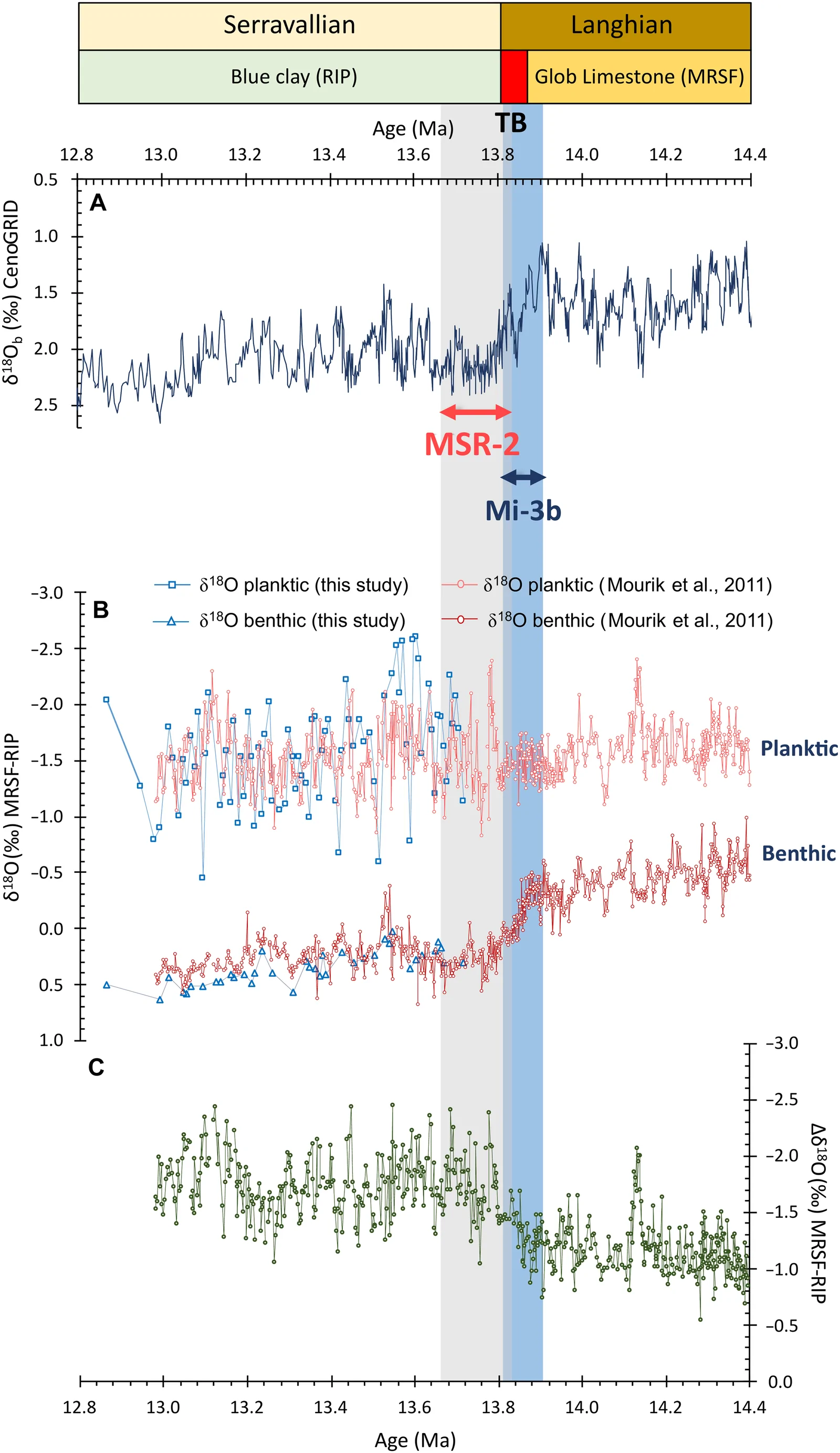
Evidence for enhanced hydroclimatic activity in the Central Mediterranean following the Middle Miocene expansion of the Antarctic ice sheet. (**A**) The global benthic δ^18^O records (*4*) (**B**) Previously published (*9*) and new records presented in this study from the Malta planktic δ^18^O records show variability following Mi-3b that is not found in other records (**C**) this variability is clearer in the planktic-benthic gradient Δδ^18^O_p-b_ from the same records.

**Fig. 3.**
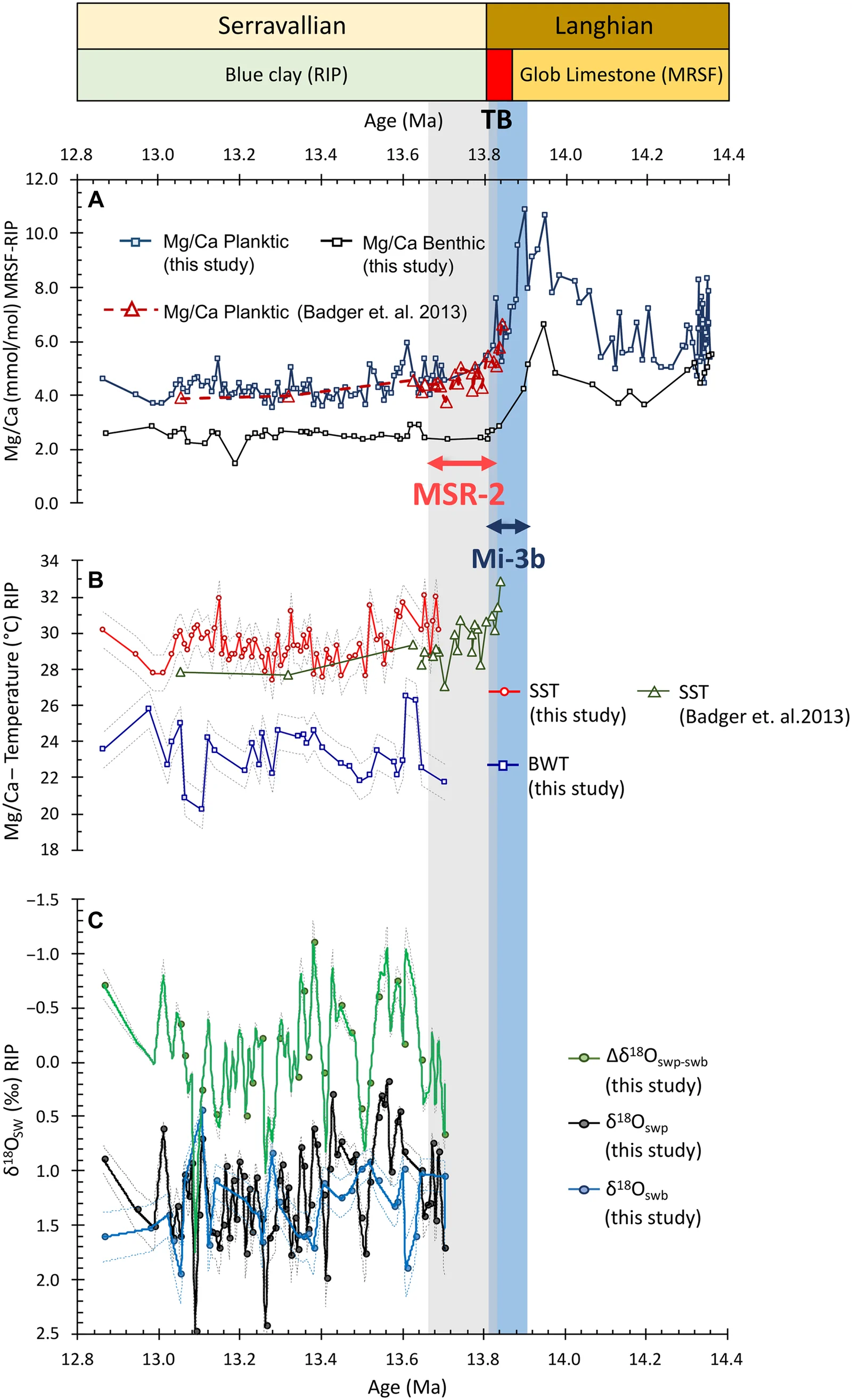
Evidence for enhanced hydroclimatic activity in the Central Mediterranean following the Middle Miocene expansion of the Antarctic ice sheet. Mg/Ca records (**A**) are here used to generate surface and bottom water temperature records (**B**). Envelopes around the temperature records indicate a sensitivity of ±1°C. (**C**) δ^18^O_sw_ of surface (δ^18^O_swp_) and bottom (δ^18^O_swb_) water have been obtained by using the Mg/Ca temperature records and their difference, Δδ^18^O_swp-swb_ is used as an indicator of evaporation/precipitation at the surface and thus acts as a proxy for salinity at the surface. Circles indicate actual available readings while the solid lines indicated linearly interpolated values. Dotted envelopes indicate uncertainties carried over from the temperature sensitivity of ±1°C (*16*).

Indeed, our calculated Δδ^18^O_swp-swb_ record has orbital scale amplitude variability of ∼1.6 ‰ following the Mi-3b excursion. We also note that the continuous Δδ^18^O_p-b_ record decreases by ∼1.8 ‰ across the MMCT, showing that the surface waters are significantly more depleted in ^16^O relative to bottom waters following the ice growth. Our results therefore allow us to be confident that the increased variability in Mediterranean planktic δ^18^O across the MMCT reflects a variable and active local hydroclimate, and that mean sea surface salinity was lower following the ice growth, during the deposition of the Blue Clay Formation. After updating the Malta age model to tune it to the CenoGRID age model, which for the Middle Miocene is solely based on data from site U1338 (*4*, *25*, *26*), we also performed spectral analysis on the previously published (*9*) δ^18^O data for the 14.4 Ma to 12.8 Ma interval, and compared it with the benthic δ^18^O record from east equatorial Pacific site IODP U1338 (*27*) (Fig. 4). This analysis shows that the precession (21 kyr) component of the benthic δ^18^O_b_ records from Malta and IODP U1338 is relatively weak throughout the entire climate transition (Fig. 4, A and B). In contrast, the precession component of the Malta planktic δ^18^O_p_ record increases in power following the expansion of the Antarctic Ice Sheet at 13.8 Ma (Fig. 4C).

**Fig. 4.**
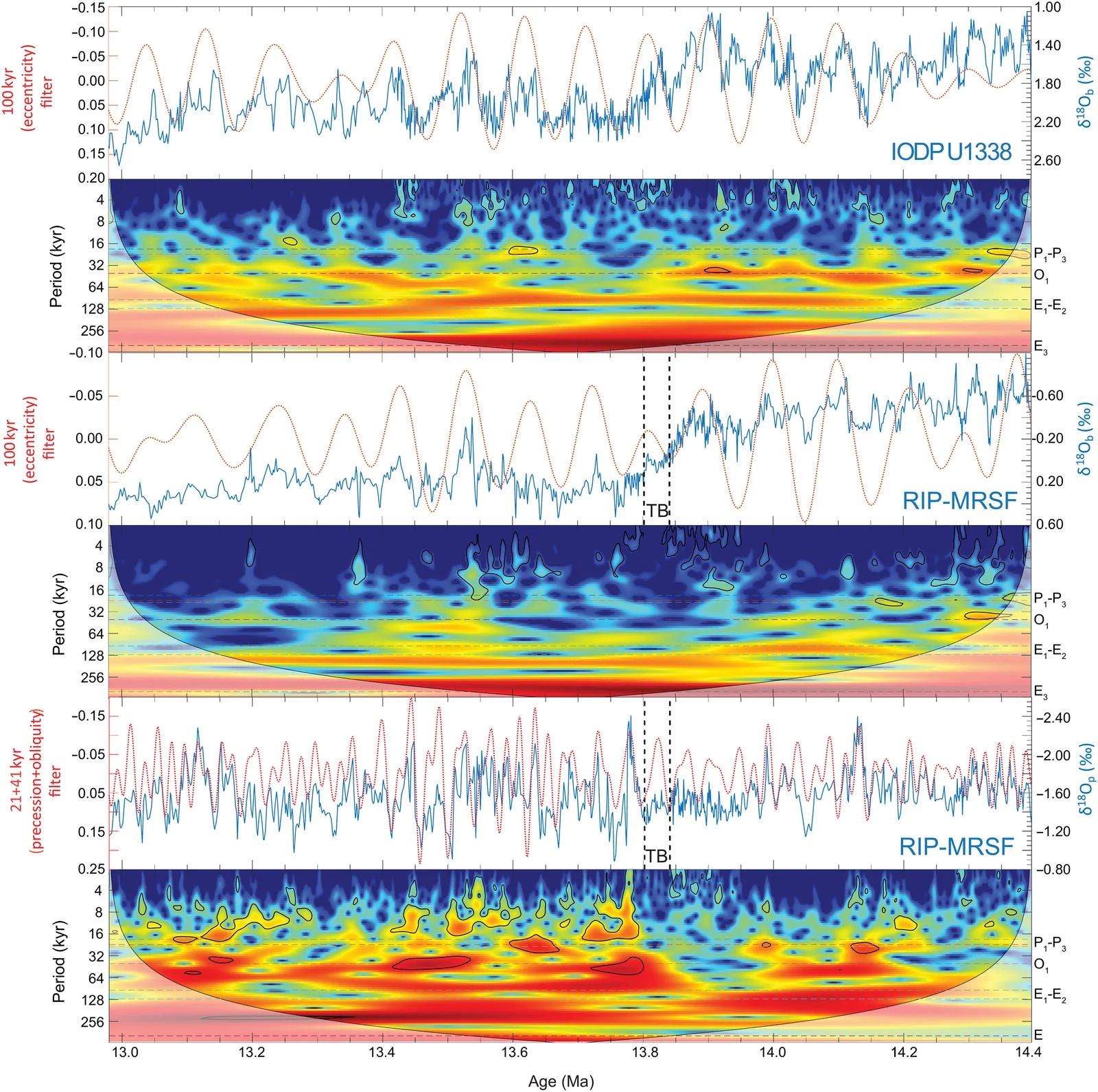
Orbital parameters for the Middle Miocene records. (**A**) CenoGRID global benthic records (*4*) which are based on U1338 (*28*) for this time interval. (**B**) Maltese benthic δ^18^O_b_ records for the composite Marsalforn (MRSF) and Ras il-Pellegrin (RIP) sites (*9*). (**C**) The planktic δ^18^O_p_ records from Malta are strongly modulated by the 21 kyr and 41 kyr obliquity and precession cycles following the transition bed (TB).

We also updated the age model for ODP site 1092 (Southern Ocean) and identified a notable misalignment in the age models presented in literature (figs. S4 and S5) (*24*, *28*). This reveals that the ODP site 1092 boron isotope based (δ^11^B) *p*CO_2_ is offset by ∼800 kyrs and does not cover the interval representing the main MMCT transition (Mi-3b excursion) or the CM-6 excursion (Fig. 5C). This result resolves the problem of the mismatch in the Malta and the Southern Ocean *p*CO_2_ records around the MMCT.

**Fig. 5.**
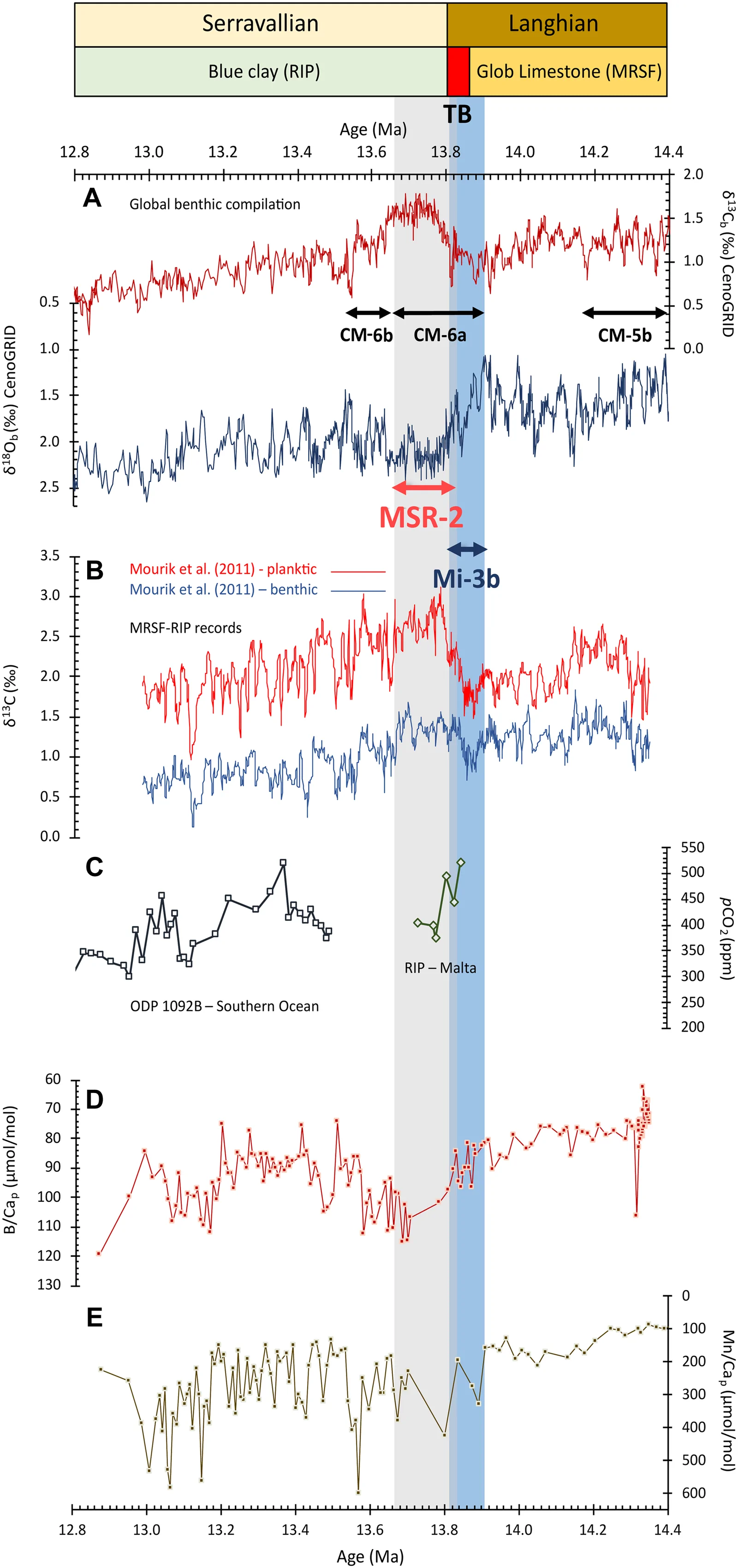
Global stable isotope records and carbon cycle indicators from Malta. (**A**) Global benthic δ^13^C and δ^18^O compilation (*4*). (**B**) Previously published planktic and benthic δ^13^C records from the Marsalforn (MRSF) and Ras il-Pellegrin (RIP) sections in Malta (*9*). Both records show the pronounced CM-5b, CM-6a and CM-6b that are prominent in the global compilation however, these are more prominent in the planktic record. (**C**) Previously available *p*CO_2_ records from around the Middle Miocene Climate Transition (*16**,* *24*). Both records have been retuned to match the CenoGRID age model. Newly obtained planktic (*Trilobatus trilobus*) trace element ratios for B/Ca (**D**) and Mn/Ca (**E**) are here presented to investigate carbon cycle dynamics in conjunction with the δ^13^C records.

### Carbon cycle and redox records across the MMCT

The newly tuned benthic and planktic δ^13^C records from Malta (*9*) both demonstrate two intervals of elevated δ^13^C which can be correlated with the global δ^13^C compilation (*4*) (Fig. 5, A and B). The earliest of these intervals (14.32 Ma to 14.14 Ma) can be correlated to the CM-5b δ^13^C positive excursion and is more prominent in the planktic than the benthic record. The younger δ^13^C excursion is also significantly more prominent in the planktic records and is correlatable with the CM-6a and CM-6b double peak positive δ^13^C excursion, confirming previous studies on the Malta stable isotope records (*9*, *16*). Following the CM-6 excursion both planktic and benthic records decrease steadily, with the planktic record demonstrating high-amplitude, high-frequency variability that is not observed in the benthic record.

Prior to the Mi-3b excursion, from 14.3 Ma to 14.0 Ma, planktic (*Trilobatus trilobus*) B/Ca_p_ concentrations vary around a mean of 83 μmol/mol (range 62 to 106 μmol/mol) after which B/Ca_p_ increases, reaching a peak of ∼116 μmol/mol at 13.7 Ma. In the earlier part of the Serravallian (13.80 Ma to 13.45 Ma), the record is persistently high (∼110 μmol/mol), prior to an interval (13.45 Ma to 13.20 Ma) of generally low values. The final part of the record (13.2 Ma to 12.8 Ma) consists of high values oscillating between ∼85 μmol/mol and ∼110 μmol/mol (Fig. 5D). Similar to the B/Ca_p_ record, the composite planktic Mn/Ca_p_ record (from the same species), shows significant differences between the Langhian and the Serravallian interval (Fig. 5E). The older part of the record consists of generally lower values, around 100 μmol/mol while the younger record displays predominantly higher values that vary between 100 μmol/mol and 600 μmol/mol. The Mn/Ca_p_ record displays high amplitude variability (∼300 μmol/mol) at high frequency (∼40 kyr) following the Langhian-Serravallian transition (13.8 Ma). In general, values oscillate between ∼100 μmol/mol and ∼380 μmol/mol for a large part of this interval (∼13.8 Ma to ∼13.2 Ma). The final part of the record (from ∼13.2 Ma to ∼12.9 Ma) displays an increasing trend with values reaching around 600 μmol/mol. The high frequency, large amplitude variability is evident throughout the whole Serravallian interval.

## DISCUSSION

### Enhanced monsoonal hydroclimate in western North Africa following the MMCT

The high-frequency variability in the planktic δ^18^O record (Fig. 2B), which follows the Middle Miocene expansion of the Antarctic Ice Sheet and the closely associated closure of the Mesopotamian Seaway, is not evident in either the Ras il-Pellegrin benthic δ^18^O (*9*) record nor the Ras il-Pellegrin bulk isotope record (*10*), nor the fine fraction (<63 μm) bulk isotope record (*16*). It is also not represented in the global δ^18^O benthic compilation (*4*) or in planktic δ^18^O records from the Southern Ocean (*21*), Kerguelen Plateau-Southern Ocean (*29*) and the Indo-Pacific region (*30*). However, we note that the records from the Indo-Pacific region are at a lower resolution than the records from Malta and so this statement needs to be interpreted with caution (Fig. 6). Our Mg/Ca-SST record does not show similar, high amplitude temperature variations. We can therefore be confident that the variability in the Maltese planktic δ^18^O record reflects high amplitude variations in sea surface salinity driven by fresh water influx (Fig. 3C). Spectral analysis of the high resolution planktic δ^18^O (*9*) (Fig. 4C) reveals a precession signal and supports a monsoonal influence on the δ^18^O variability in the planktic records. The contemporaneous shift in the Δδ^18^O_p-b_ record and the transition from carbonate-dominated deposition to clay-dominated deposition is consistent with an overall increase in monsoonal activity following the ice growth and final closure of the Mesopotamian Seaway (Figs. 1C and 2C). The maximum variability in Δδ^18^O_swp-swb_ (∼1.7 ‰) following the Mi-3b excursion and MSR-2 (Fig. 3C) is comparable with negative excursions in seawater δ^18^O (0.5 to 3.0 ‰) associated with Pleistocene monsoonal activity and sapropel deposition in the Eastern Mediterranean that were driven by bursts of freshwater from the Nile (120 ka to 350 ka) (*31*). It is most likely that fresh water and clastic material (source material for the Blue Clay Formation, Fig. 1C) was supplied via strong fluvial systems that periodically discharged in the Gulf of Gabes (Tunisia) as proposed for the Early Miocene post MSR-1 (∼20 to 19 Ma) Central Mediterranean humid interval (*12*, *13*).

**Fig. 6.**
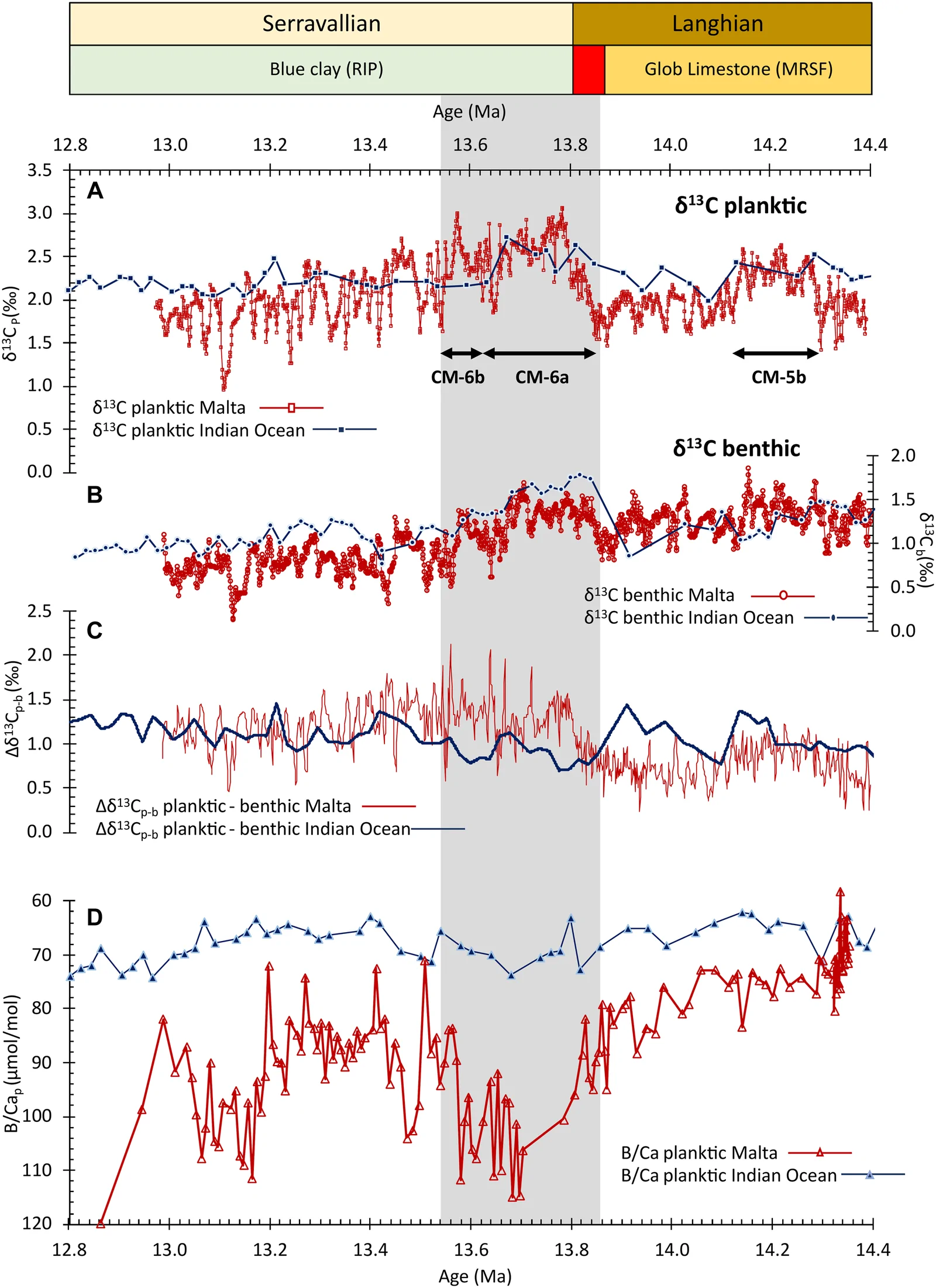
Comparison between shelf environment (Malta) with the open ocean (ODP 761). (**A**) Planktic and (**B**) benthic δ^13^C records from ODP 761 (Indian Ocean) (*30*) and from Malta. (**C**) The δ^13^C gradient (Δδ^13^C_p-b_) from both sites. (**D**) The distinction between the planktic B/Ca from the two sites. Gray bar represents the CM-6 excursion.

Changes in mineralogy and bulk carbonate δ^18^O from Malta have been used to speculate that Central Mediterranean climate became more humid during the Middle Miocene, perhaps caused by an enhanced thermal gradient between the tropics and the South Pole and an ensuing northward shift of climate belts including the Intertropical Convergence Zone (*6*). Our new records allow for precise correlation between the MMCT expansion of the Antarctic Ice Sheet and geochemical records from Malta, including hydrological change and gateway closure.

The Middle Miocene expansion of the Antarctic Ice Sheet occurred in three steps (*3*) with a large step starting at 13.9 Ma coinciding with the transition bed (TB) that preceded deposition of the Blue Clay formation in Malta (Fig. 1) (*9*, *10*, *16*, *17*, *24*). Measurement of the εNd tracer from the Maltese Globigerina Limestone and Blue Clay formation (*2*) clearly shows that the Antarctic Ice Sheet expansion and ensuing sea-level fall (∼ 50 m) (*32*) closely coincided with the seaway closure (MSR-2) (Figs. 2 and 3). Therefore, this final disconnection between the Indo-Pacific realm and the Atlantic realm via the Mediterranean was most likely driven by shoaling due to the eustatic sea level fall associated with the expansion of the Antarctic Ice Sheet. Fully coupled ocean-atmosphere general circulation models suggest that the Mesopotamian Seaway closure had important global climatic impacts (*33*). These models show that an open Mesopotamian gateway advects hot, dense Tethyan Intermediate Sea Water (TISW) into the Indian Ocean. The absence of TISW in the Indian Ocean has the tendency of generating a greater latitudinal density gradient which strengthens the Antarctic Circumpolar Current (ACC), potentially thermally isolating Antarctica (*33*). The Mesopotamian Seaway closure would have also resulted in the advection of hot, dense Mediterranean intermediate waters via the proto-Strait of Gibraltar resulting in intensification of the Atlantic Meridional Overturning Circulation (AMOC) (*2*, *34*). This can in turn enhance the ACC by generating a stronger latitudinal pressure gradient in the Atlantic Ocean (*33*). It can also affect the regional land-sea temperature contrast in the Atlantic basin, directly impacting the West African Monsoon System (*35*). The earlier (∼19 Ma) (MSR-1) tectonically-driven restriction of the Mesopotamian Seaway occurred during a warming interval, and is therefore thought to have enhanced the West African Monsoon system by changing the regional land-sea temperature contrast in the Atlantic basin (*13*). The final closure of the Mesopotamian Seaway during the Middle Miocene (MSR-2) was most likely precisely synchronous with the Antarctic Ice Sheet expansion. It is therefore difficult to disentangle the relative roles of global latitudinal temperature gradients and regional land-sea temperature contrasts in driving the Middle Miocene transition to a more monsoonal type climate.

### Carbon cycle impacts of enhanced monsoonal activity

Enhanced hydrological activity increases chemical weathering rates with monsoon activity being the main factor that influences weathering at low latitudes in Asia today (*36*, *37*). Orbitally controlled nutrient delivery by river fluxes is considered to have a very important role in enhancing productivity and carbon burial (*37*). It can be very reasonably assumed that the data presented here represents enhanced humidity of the Sahara which had a pronounced effect in triggering river systems in present day Tunisia and Libya. Such systems are thought to have been periodically activated throughout the Miocene (*18*) and would have resulted in enhanced nutrient delivery into the Central Mediterranean. This is somewhat similar to what has been previously proposed for the Early Miocene (*12*, *13*). The planktic and benthic Malta δ^13^C records display similar trends to the global compilation (*4*) (Fig. 5, A and B). However, the high frequency δ^13^C variations in the planktic record from Malta are more amplified than both the Malta benthic record (Fig. 5B) and typical open ocean planktic records such as ODP Site 761 (Fig. 6A). For CM-6 in particular, it is difficult to explain the magnitude of the Malta planktic positive δ^13^C shift through a reduction in air-sea gas exchange, when sea surface temperatures in this region were falling (*16*) and there is no evidence for cessation of upwelling. Therefore, the most likely explanation is that the amplified planktic δ^13^C signal is reflecting a rapid increase in productivity in the Central Mediterranean surface waters that starts at 13.86 Ma coinciding with the onset of CM-6. We test this interpretation using our geochemical records.

The surface to seafloor δ^13^C gradient (Δδ^13^C_p-b_) is used here to assess changes in the efficiency of the biological pump (*38*). At Malta, Δδ^13^C_p-b_ increases by ∼1.3 ‰ across the CM-6 interval and remains relatively high for the remaining studied interval suggesting an increase in biological pump efficiency (Fig. 6C). We also calculate Δδ^13^C_p-b_ from Indian Ocean Site ODP 761 (*39*) and unlike Malta, Δδ^13^C_p-b_ at ODP Site 761 decreases across CM-6, because the planktic δ^13^C signal is muted compared with the benthic signal at this open ocean site (Fig. 6). This shows that the increase in export productivity associated with CM-6 in Malta represents a regional signal. It may be argued that Δδ^13^C_p-b_ is not a reliable biological pump proxy in a region that might have experienced changes in advection, and therefore we also investigate productivity changes using our trace metal records.

The generally low planktic foraminiferal Mn/Ca_p_ values (<100 μmol/mol) prior to the Mi-3b excursion suggest relatively low levels of authigenic Mn-coatings (Fig. 5E) (*40*). The Mn/Ca values following Mi-3b mostly exceed 100 μmol/mol, indicating that more Mn is present in coatings on the rough *T. trilobus* test. This can be taken as evidence of low oxygen conditions within the sediment. Low oxygen conditions mobilise Mn^2+^ which becomes available for incorporation into manganese phases on the *T. trilobus* test (*41*). A low oxygen environment in the shallow subsurface is consistent with a highly active biological pump in the region. The higher abundance of the benthic foraminifera *Bulimina elongata* (*15*) in the Blue Clay Formation relative to the Globigerina Limestone Formation at Ras il-Pellegrin also indicates low oxygen benthic environment suggestive of a highly active biological pump following the intensification of monsoonal activity in the region. Taken together, the evidence suggests that the high amplitude changes in the planktic δ^13^C and Δδ^13^C_p-b_ in Malta reflect orbitally paced episodes of enhanced export productivity. It is therefore likely that the intervals of intense export productivity in the Central Mediterranean were driven by orbitally paced intervals of enhanced freshwater discharge and associated nutrient delivery following the Middle Miocene expansion of the Antarctic Ice Sheet and the MSR-2 event. This can be shown by noting significant correlation between positive excursions in the δ^13^C_p_ and negative excursions in the δ^18^O_p_ records from Malta following these events (Fig. 7A). The local impact of the enhanced biological pump on seawater carbonate chemistry is assessed by comparing the B/Ca_p_ records from Malta with records from the same planktic species from ODP Site 761 (Fig. 6D) (*30*, *42*).

**Fig. 7.**
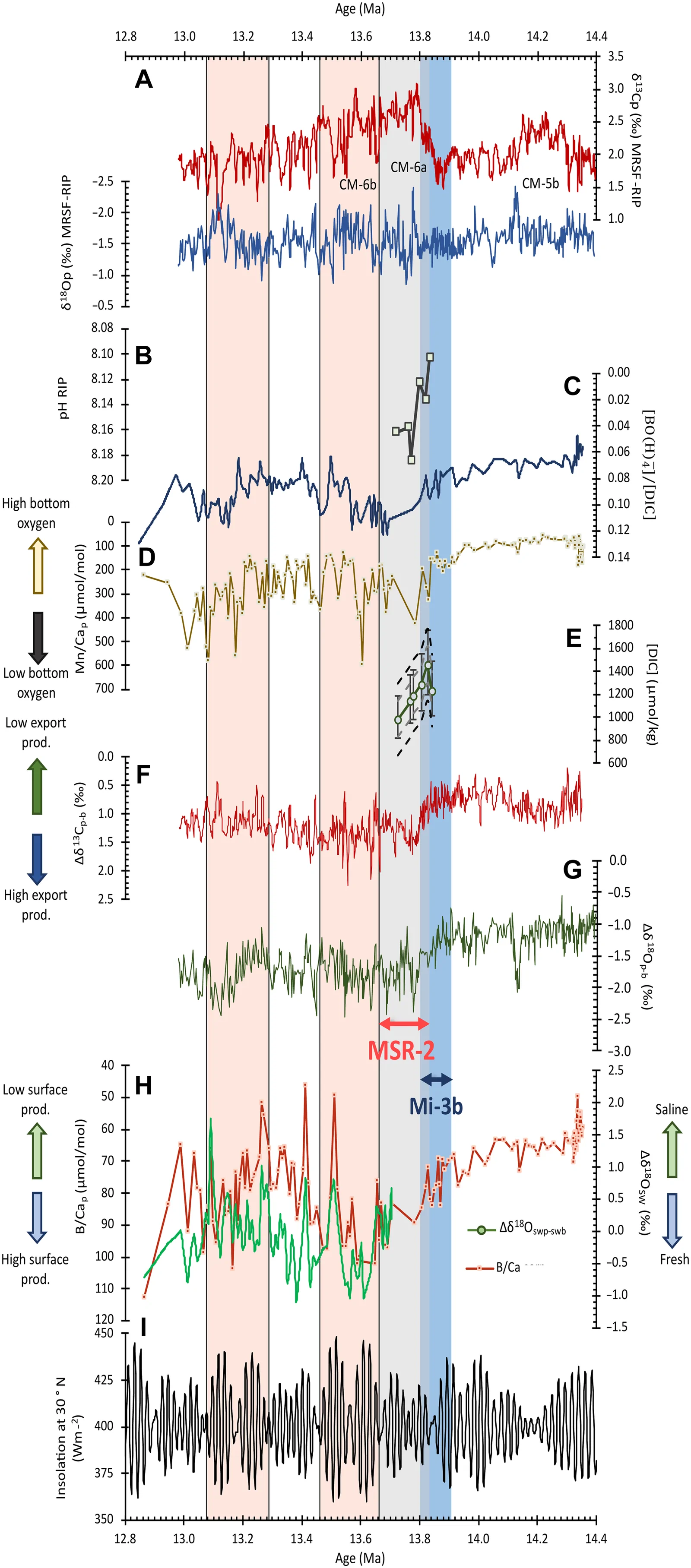
Carbon cycle and climate following the Middle Miocene expansion of the Antarctic ice sheet. Planktic stable isotope (δ^18^O and δ^13^C) records from Malta (**A**). (**B**) Previously published pH records from Malta (*16*)_._ (**C**) $\left[\text{BO}{\left(H\right)}_{4}^{-}\right]/\left[\text{DIC}\right]$ from the B/Ca records compared with Mn/Ca (**D**) and calculated DIC records (**E**) for interval for which pH values from Ras il-Pellegrin are know. Light grey dotted envelope represents ±1 SD, black dotted envelope represents ±2 SD assuming a normal distribution of data. Error bars obtained by considering a sensitivity of ±0.1 on the pH values of Badger *et al.* (*16*) (**F**) Δδ^13^C_p-b_ from Malta as a proxy for biological pump efficiency in the Central Mediterranean during the Middle Miocene. Δδ^18^O_p-b_ (**G**) showing tight coupling with B/Ca and Δδ^18^O_swp-swb_ (surface salinity proxy) (**H**). Increases in B/Ca associated with lowering of salinity and hence inputs of fresh water and nutrients into the Central Mediterranean. Maximum variability in the B/Ca, Δδ^18^O_p-b_, Δδ^18^O_swp-swb_, Mn/Ca, Δδ^13^C records coinciding with times of high insolation variability (**I**) (coral bars) at low latitudes.

The records show that the Central Mediterranean experienced a marked change in carbonate chemistry that is not observed at ODP Site 761. *Trilobatus trilobus* B/Ca_p_ and hence reconstructed surface $\left[B{\left(\text{OH}\right)}_{4}^{-}\right]/\left[\text{DIC}\right]$ are persistently low during the earlier part of the record (pre Mi-3b; Fig. 7, C and H). Since the Central Mediterranean pH record does not cover this earlier pre CM-6 interval it is impossible to disentangle the [DIC] vs $\left[B{\left(\text{OH}\right)}_{4}^{-}\right]$ contributions (Fig. 7, B and C to E). Persistently low *T. trilobus* Mn/Ca (∼100 μmol/mol to 200 μmol/mol) suggests limited authigenic manganese carbonate coatings and a well oxygenated seafloor during this time (Fig. 7D). This is consistent with relatively low export productivity, which would tend to increase surface [DIC] and lower $\left[B{\left(\text{OH}\right)}_{4}^{-}\right]$, as shown by the record presented here (Fig. 7C). These records perhaps suggest that export productivity in the Central Mediterranean during the CM-5b carbon maximum was not particularly different from the open ocean (Fig. 6C).

*Trilobatus trilobus* B/Ca_p_ and Mn/Ca_p_ gradually increase from ∼14.1 Ma (Fig. 7, D and H), in the interval immediately preceding the main ice growth episode of the MMCT. The pH data from the Ras il-Pellegrin section (*16*) allow for the determination of [DIC] during this phase and also for CM-6 (Fig. 7E). Declining [DIC] and increasing pH are consistent with declining CO_2_ as the primary mechanism for causing the MMCT, as previously suggested (*5*). *Trilobatus trilobus* B/Ca_p_ and Mn/Ca_p_ increase further around 13.9–13.8 Ma, associated with the onset of intense monsoonal activity, a change in lithology, and an increase in Δδ^13^C_p-b_. Our new multi-proxy records therefore indicate that the wetter conditions following the Mi-3b expansion of the Antarctic Ice Sheet led to significantly increased export productivity in the Mediterranean region. It is likely that regional export productivity was highly variable because the monsoon strength would have been very sensitive to precessional forcing. To investigate this idea, we compare the planktic B/Ca_p_ with the Δδ^18^O_p-b_ and Δδ^18^O_sw_ records (Fig. 7, G and H). High salinity tends to increase foraminiferal B/Ca (*42*) but surface productivity is expected to increase during phases of low salinity when the influx of fresh water from western North Africa also delivers nutrients to surface waters. Furthermore, culture experiments indicate that $\left[B{\left(\text{OH}\right)}_{4}^{-}\right]/\left[\text{DIC}\right]$ changes by ∼0.001 for a change in salinity of 1 PSU and B/Ca increases by ∼6 μmol/mol for the same salinity increase (*42*). These changes are very small compared with the magnitude of changes in our B/Ca_p_ and $\left[B{\left(\text{OH}\right)}_{4}^{-}\right]/\left[\text{DIC}\right]$ records. It can therefore be argued that variations in sea surface salinity only serve to slightly dampen the productivity signal in the foraminiferal B/Ca record. Our records support our prediction that regional productivity also varied significantly on orbital timescales (Fig. 7H). Changes in the $\left[B{\left(\text{OH}\right)}_{4}^{-}\right]/\left[\text{DIC}\right]$ data for the Ras il-Pellegrin section are as large as 0.06 in certain parts of the record particularly during the earliest (∼13.66 Ma to ∼13.46 Ma) and latest (∼13.28 Ma to ∼13.08 Ma) parts (Fig. 7C). These also correspond to the parts of the record with the largest swings in Δδ^18^O_swp-swb_ and with lowest inferred salinity values (and hence highest fluxes of fresh water and nutrients). Therefore, enhanced export productivity in the Central Mediterranean during CM-6 was likely caused by monsoonal activity in North Africa in a cooling world. This may explain why no strong productivity signals are observed in this region during CM-5b, indicating limited monsoonal activity over North Africa at a time with an intermittently open Mesopotamian Seaway and lower latitudinal temperature gradients of the earliest part of the MMCT.

### Interpretation of CM-6 and global CO_2_ following Middle Miocene ice growth

CM-6 is the final and largest carbon maxima event within the Monterey Carbon Isotope Excursion and is associated with the Mi-3b ice growth (*43*). The overall positive Monterey Carbon Isotope Excursion is now thought to represent high DIC in a warm greenhouse world (*44*). However, different mechanisms have been proposed to explain the individual orbitally-paced CM events. Planktic B/Ca and δ^13^C records were used to suggest that these reflect intervals of enhanced organic carbon burial that removed ^12^C-enriched carbon from the ocean-atmosphere system while drawing down atmospheric CO_2_ (*45*). An alternative proposed explanation for the long eccentricity δ^13^C cycles in the Middle Miocene is enhanced deposition of shelf carbonates with positive δ^13^C signatures during sea level highstands (*44*). In this scenario, CM-6 would be caused by a glacio-eustatically induced decrease in shallow water carbonate deposition, and associated with decreasing CO_2_ and enhanced preservation of CaCO_3_ in the deep sea (*44*).

The first CO_2_ reconstruction through CM-6 used samples from Malta and was consistent with these interpretations, with boron isotope and alkenone based proxies both showing a decline in CO_2_ through the CM event (*16*). However, the boron isotope-based CO_2_ record from the Southern Ocean (ODP Site 1092) appeared to indicate an increase in atmospheric *p*CO_2_ during the CM-6 carbon-isotope excursion (*24*). This was interpreted as an impact of enhanced ocean ventilation, with the implication that the Malta record did not represent global trends (*24*). However, the revaluation of the age model for ODP 1092 presented here shows that the records from the Southern Ocean are younger than the data from Malta. Thus, it seems that the only high-resolution CO_2_ record through CM-6 (*16*) supports a sustained global decrease in *p*CO_2_ following the Middle Miocene glaciation.

We also note that the shelf carbonate model alone is unable to simulate the full magnitude of the CM-6 excursion (*44*) and suggest that the unusual magnitude of CM-6 requires the additional mechanism of enhanced productivity and associated carbon burial (*45*). Our records indicate that the Mediterranean region could have contributed to this δ^13^C excursion and associated CO_2_ drawdown. One important implication of this conclusion is that the Malta records reflect such a strong local productivity signal that it is unclear to what extent the Maltese *p*CO_2_ records reflect a global signal. We therefore recommend that a high resolution CO_2_ record through CM-6 is reconstructed at an open ocean oligotrophic site.

### Regional hydrodynamics and their impact on paleo *p*CO_2_ records

By combining planktic and benthic stable isotope and multi-proxy trace metal records from well preserved Maltese Miocene foraminifera, we have shown that the expansion of the Antarctic Ice Sheet and the associated glacioeustatic closure of the Mesopotamian Gateway that swiftly followed had a profound influence on the hydroclimate and oceanography of the Mediterranean region. Our records demonstrate the presence of orbitally paced episodes of monsoonal activity and hence humidification of Western Sahara. The resultant nutrient-driven increase in biological productivity had a marked effect on the regional carbon cycle and sequestration of organic carbon in seafloor sediments. It is possible that such high-productivity sites represented important positive feedback in the global carbon cycle. Yet, high-resolution records across the whole MMCT and associated CM-6 δ^13^C excursion from different sites are still non-existent. In the absence of such records that would allow detailed comparison between different sites governed by notably different paleoenvironments it is impossible to weigh the different contributions of the various feedbacks to the carbon cycle. The records from Malta offer a compelling history highlighting the complex interplay between gateway restriction, global climate, and regional humidity as Earth transitioned from the relatively warm Miocene Climatic Optimum to a world with a large Antarctic Ice Sheet. This study confirms the importance of regional influences on marine CO_2_ proxies, but also highlights the relatively untapped potential for understanding complex carbon cycle processes through careful site selection.

## MATERIALS AND METHODS

### Study sites and age model

The dry samples from two Maltese sections in Marsalforn (Island of Gozo 36°04.24’N 14°15.51E) and Ras il-Pellegrin (Main island, 35°54.93’N 14°20.06′E) were obtained from previous field expeditions (*9*, *16*). Samples from both sections were used to produce one continuous composite stratigraphy.

A new age model for the two composite sections was produced by tying points (Supplementary Material sec. 1) using a combination of high resolution bulk stable isotope records (δ^18^O and δ^13^C) measured during this study, previously available bulk stable isotope records (δ^18^O and δ^13^C) (*10*, *16*) and, benthic foraminifera δ^18^O stable isotope records (*9*). These records were aligned to the Cenozoic Global Reference benthic foraminifer carbon and oxygen Isotope Dataset (CenoGRID) (*4*). This new age model for the Marsalforn and Ras il-Pellegrin sections differs from the previously published models (*9*, *16*) in that it does not pre-assume an astronomical (precession ∼20 kyr) forcing frequency on the record. This allows for identifying the spectral components in the various proxies used in this study without running into circular arguments.

The δ^11^B record for ODP 1092 (*24*) has also been aligned with CenoGRID. This has been achieved using the available magnetostratigraphy (*46*, *47*) and stable isotope (δ^18^O and δ^13^C) (*28*) records from ODP 1092. We notice a misalignment between the depth records and the magnetostratigraphy in the age model presented by Kuhnert and co-authors (2009) (*28*). Specifically, the alignment between the depth and magnetic chron is wrong (Table S2). Unfortunately this error in alignment between depth and magnetic polarity was propagated in the age model of Raitzsch and co-authors (2021) (*24*). We made use of the original magnetostraitgraphy for ODP 1092 (*47*) in order to fix this problem and align the age model for ODP 1092 to the CenoGRID (*4*) age model. For the interval between chron C5Ar.1n (top) and C5Bn (base), a total of 7 CenoGRID tie points were utilised (*46*, *47*) [table S2 and fig. S5 (*46*)]. The revised age model was then applied to the available stable isotope records (δ^18^O and δ^13^C) (*28*) and aligned with the δ^18^O and δ^13^C from U1338 (CenoGRID) in order to verify the age model (fig. S4).

### Stable isotope and trace metal records from foraminifera

Samples were dry sieved through a 355, 250, 100 and 63 μm sieve stack. Planktic and benthic foraminiferal species were picked from the 250 to 355 μm size fraction. Selection of species was carried out according to abundance of specimens in the samples. The selected planktic species was *Trilobatus trilobus* while two benthic species *Uvigerina* spp. and *Heterolepa dutemplei* were selected due to lack of abundance of benthic specimens. Specimens of *Trilobatus trilobus* and *Uvigerina* spp. were prepared for trace metal analysis while specimens of *Trilobatus trilobus* and *Heterolepa dutemplei* were prepared for stable isotope analysis.

Stable isotope (δ^18^O and δ^13^C) analysis was carried out on benthic and planktic specimens from the Ras il-Pellegrin section. These were compared with previously available stable isotope records from the same site (*9*). Stable isotope analysis was carried out at the School of Earth and Environmental Sciences, Cardiff University. 3 to 4 specimens of the benthic species *Heterolepa dutemplei* were used while 5 to 7 specimens of the planktic species *Trilobatus trilobus* were utilized (total carbonate material ∼88 μg to 120 μg). Results were expressed in ‰ in the δ notation on the Vienna Pee Dee Belemnite (VPDB) scale. Analysis was carried out on MAT 253/Kiel IV carbonate device. Long term precision of in-house standard (BCT63) is ≤0.04 ‰ (1 s.d.) in δ^18^O and ≤ 0.03 ‰ (1 SD) in δ^13^C.

For trace metal analysis 25 to 30 specimens of the planktic species *T. trilobus* (∼440 μg to 530 μg of total carbonate material) and 10 to 15 samples of *Uvigerina* spp. were cracked open between two clean glass slides following an in-house modified method (*48*). Foraminifera chambers were opened and inspected under light microscope and infill material (clay, pyrite) was gently removed using a fine brush. The foraminiferal fragments were set aside, dried using a dry brush and scooped into a labelled, acid-cleaned 500 μl centrifuge tube. A total of 84 samples containing planktic foraminifera from the RIP section and 68 from the MRSF section were utilized. A total of 54 samples of benthic foraminifera were utilized, 34 from the RIP section and 20 from the MRSF section. Where available, planktic Mg/Ca data was compared with previously available data from (*17*) the same site and same species (*16*).

The crushed foraminiferal samples were cleaned under clean laboratory conditions at The School of Earth and Environmental Sciences, Cardiff University (*48*). The cleaned and dry samples were dissolved in 120 μl of ultra-pure 0.065 M HNO_3_. The dissolution process was aided by placing each tube on a vortex mixer. The solutions were placed in an ultra-centrifuge and centrifuged at 30,000 rpm. An aliquot of 10 μl from the centrifuged solution was gently pipetted and placed in a clean centrifuge tube to be diluted and used for calcium analysis while an aliquot of 100 μl was pipetted and placed in a clean tube to be diluted and used for trace element analysis. Trace element and calcium analysis were carried out using a Thermo Element XR High Resolution – Inductively Coupled Plasma – Mass Spectrometer (HR-ICP-MS) at the Cardiff Earth Laboratory for Trace Element and Isotope Chemistry (CELTIC) at Cardiff University. Long-term precision for trace element to calcium ratios: Mg/Ca ≤ 1%, B/Ca ≤ 2%, Mn/Ca ≤ 1%, Sr/Ca ≤ 1%, Ba/Ca ≤ 4%, Fe/Ca ≤ 4%, U/Ca ≤ 2%.

Intact and crushed planktic (*T. trilobus*) and benthic (*Uvigerina* spp.) specimens from the RIP and MRSF sections were used to generate Scanning Electron Microscope (SEM) images. The fragments were cleaned with DI water and acetone using fine paint brushes in order to remove any infill material. Prior to imaging, the samples were mounted and coated with gold and palladium. Imaging was performed using a FEI XL 30 Field Emission Gun Environmental Scanning Electron Microscope in high vacuum mode with a beam voltage between 10 to 20 kV using a Secondary Electron Detector at the School of Earth and Environmental Sciences, Cardiff University.

### Mg/Ca paleotemperature and seawater δ^18^O calculations

Sea-surface temperatures (SST) (Fig. 3B) were calculated using a calibration for the modern day species *Trilobatus sacculifer* (*30**,* *45*) since *Trilobatus trilobus* is the morphotype for *T. sacculifer* (*49*). Calculations were performed using Eq. (1) (*50*) relating the Mg/Ca of the calcite shell of foraminifera (Mg/Ca_f_) to the sea-surface temperature, T

$$\text{Mg}/{\text{Ca}}_{f}={\left[\frac{\text{Mg}/{\text{Ca}}_{\text{sw}}\;\left(t\right)}{\text{Mg}/{\text{Ca}}_{\text{sw}}\;\left(0\right)}\right]}^{C}\;{\mathrm{Be}}^{\mathrm{AT}}$$ (1)

Where Mg/Ca_sw_ (*t*) represents the past seawater Mg/Ca ratio at age *t* and Mg/Ca_sw_(0) is the modern seawater Mg/Ca ratio of 5.2 mol/mol (*30*). For *T.trilobus* the constants used are A = 0.09, B = 0.347, C = 0.41 (*51*, *52*). The value of Mg/Ca_sw_ (t) was calculated following (*30*). Mg/Ca_sw_ does not vary significantly (from 3.6 mol/mol to 3.7 mol/mol) throughout the interval and therefore it impacts absolute temperature and salinity values but has no significant bearing on the temperature and salinity variations throughout the record. No correction for pH was conducted as it has been shown that *T. trilobus* Mg/Ca is insensitive to pH variability (*53*).

BWT values (Fig. 3B) were also calculated using Eq. (1) (*50*). The values B = 0.98 and A = 0.045 were chosen for *Uvigerina* spp. specimens that have not been subjected to a reductive cleaning method (*54*). The power constant C is unknown for *Uvigerina* spp. and C = 0.27, the value determined for the semi-infaunal species *O. umbonatus* was assumed (*50*). However, confidence in the absolute values of BWT is low and further studies would be required to obtained a higher confidence in these records.

The δ^18^O_sw_ for surface waters was determined using a calibration between the δ^18^O_f_ and the Mg/Ca surface temperature (*55*) (Fig. 3C). The bottom water δ^18^O_sw_ was obtained using a calibration between the bottom temperature Mg/Ca and the benthic δ^18^O_f_ (*56*) (Fig. 3C). For *Uvigerina* spp., the value of 0.47 ‰ was first subtracted from the δ^18^O_f_ before applying the equation. This is because *Uvigerina* spp. is isotopically heavier by 0.47 ‰ than the species *Cibicidoides* and *Planulina* (*56*).

The difference between the surface and bottom δ^18^O_sw_ is expressed as Δδ^18^O_swp-swb_ and can be considered to act as a proxy for the salinity at the surface. Since the benthic record is at a lower resolution than the planktic record, the two δ^18^O_sw_ records were first linearly interpolated and then Δδ^18^O_swp-swb_ was obtained. The sparse Δδ^18^O_swp-swb_ record obtained using only points for which coinciding planktic and benthic data is available was also obtained and plotted over the interpolated records (Fig. 3C). Considering a temperature sensitivity of ±1°C leads to an estimated uncertainty of ±0.23 ‰ in the δ^18^O_sw_ records, which propagates to ∼ ±0.33 ‰ for the Δδ^18^O_swp-swb_ records.

### Paleoproductivity proxies

The surface to seafloor δ^13^C gradient (Δδ^13^C_p-b_) is here used as a proxy for the efficiency of the biological pump (*38*). Δδ^13^C were also calculated from Middle Miocene data from ODP Site 761 (*39*) and were compared to the dataset from Malta (Fig. 6). Comparisons between the planktic B/Ca_p_ Malta records from this study and planktic B/Ca_p_ from ODP 761 (*30*) were also made (Fig. 6D). These comparisons were made on the same planktic foraminifer species, *Trilobatus trilobus*.

The linear relationship between Eq. 2 foraminifera B/Ca_f_ and $\left[B{\left(\text{OH}\right)}_{4}^{-}\right]/\left[\text{DIC}\right]$ obtained from culture experiments on *T. sacculifer* (*42*) was used to calculate variations in $\left[B{\left(\text{OH}\right)}_{4}^{-}\right]/\left[\text{DIC}\right]$ (*45*).

$$\begin{array}{cc} B/{\text{Ca}}_{f}=\frac{B/{\text{Ca}}_{\text{sw}}\;\left(t=t\right)}{B/{\text{Ca}}_{\text{sw}}\;\left(t=0\right)}\times \frac{\left[B{\left(\text{OH}\right)}_{4}^{-}\right]}{\left(\text{DIC}\right)}773+33 & R^{2}=0.81 \end{array}$$ (2)

The ratio B/Ca_sw_ (t = t) represents the B/Ca ratio of seawater at the age of the sample while B/Ca_sw_ (t = 0) is the ratio for modern day seawater. The value of B/Ca_sw_ (t = t) between 14.35 Ma and 12.86 Ma varied from 70.01 μmol/mol to 68.98 μmol/mol (*45*) and B/Ca_sw_ (t = 0) is equal to 59.88 μmol/mol (*45*). These ratios have been calculated from boron isotope modelling and fluid inclusion estimates (*45**,* *57*).

The previously available pH record from the Ras il-Pellegrin section (*16*) is lower resolution that the B/Ca_p_ record from this study and therefore the estimated [DIC] is of low resolution and cannot be used to discuss the whole B/Ca_p_ record. However, it can be used to estimate [DIC] across the main step of the MMCT (13.94 Ma to 13.66 Ma). To calculate [DIC], the pH values were first used to determine the concentration of [H+] ions Eq. 3, which was then used to calculate the borate ion concentration Eq. 4. The [DIC] was then determined from the $\left[B{\left(\text{OH}\right)}_{4}^{-}\right]/\left[\text{DIC}\right]$ ratio using Eq. 2.

$$\text{pH}=-{\text{log}}_{10}\;\left[H^{+}\right]$$ (3)

$$B{\left(\text{OH}\right)}_{4}^{-}=\frac{B_{\mathrm{TOT}}}{\left(1+\frac{H^{+}}{K_{B}^{*}}\right)}$$ (4)

The stochiometric equilibrium constant for boric acid $K_{B}^{*}$ has a value of 2.5 × 10^−9^ (*58*).

## Supplementary Materials

### This PDF file includes:

Sections 1 to 3 Figs. S1 to S10 Tables S1 to S3
