# Supplementary material for: Humid periods over western Sahara during the Middle Miocene Climate Transition and their implications on the carbon cycle
Source: Sci Adv. 2026 Nov 6;12(45):eaec7963. doi: 10.1126/sciadv.aec7963 (PMC13742176; doi:10.1126/sciadv.aec7963)
Supplement: Supplementary file 1 [file sciadv.aec7963_sm.pdf]

Science Advances
AAAS

Supplementary Materials for

Humid periods over western Sahara during the Middle Miocene Climate Transition and their implications on the carbon cycle

Ray Zammit *et al.*

Corresponding author: Ray Zammit, rzammit@geomar.de

Sci. Adv. **12**, eaec7963 (2026)
DOI: 10.1126/sciadv.aec7963

This PDF file includes:

Sections 1 to 3
Figs. S1 to S10
Tables S1 to S3

Section 1 – Age Model and stratigraphy

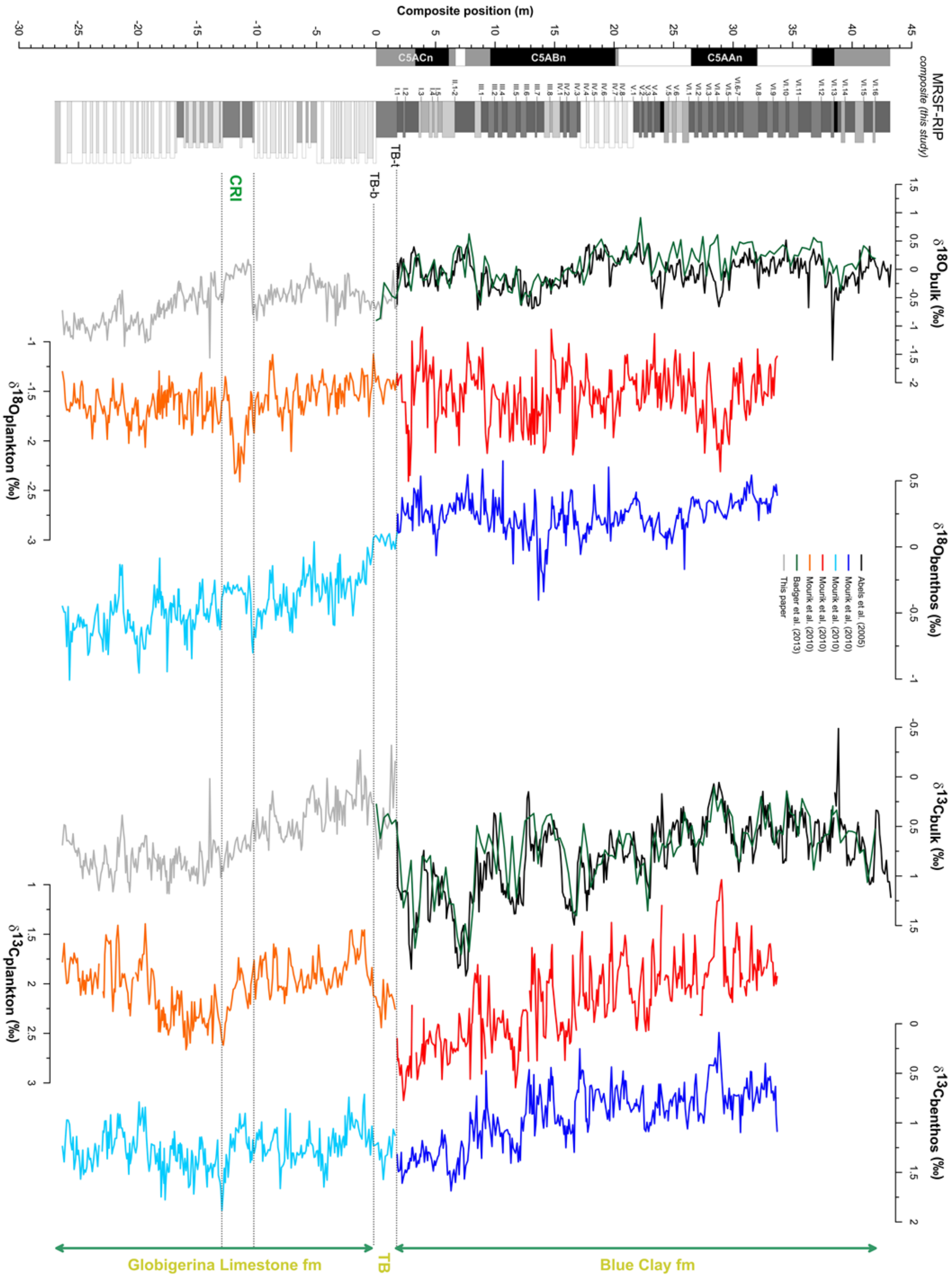

Figure S1: Carbonate stable isotope stratigraphy for the composite Marsalforn and Ras il-Pellegrin (MRSF-RIP) sections, Malta. Stable isotope data plotted with respect to the lithology of the Composite section (*9, 10, 16*). CRI – Clay rich interval (*6*), TB – transition bed between the Globigerina Limestone Formation and the Blue Clay formation (*9*). TB-b – Base of transition bed, TB-t – Top of transition bed.

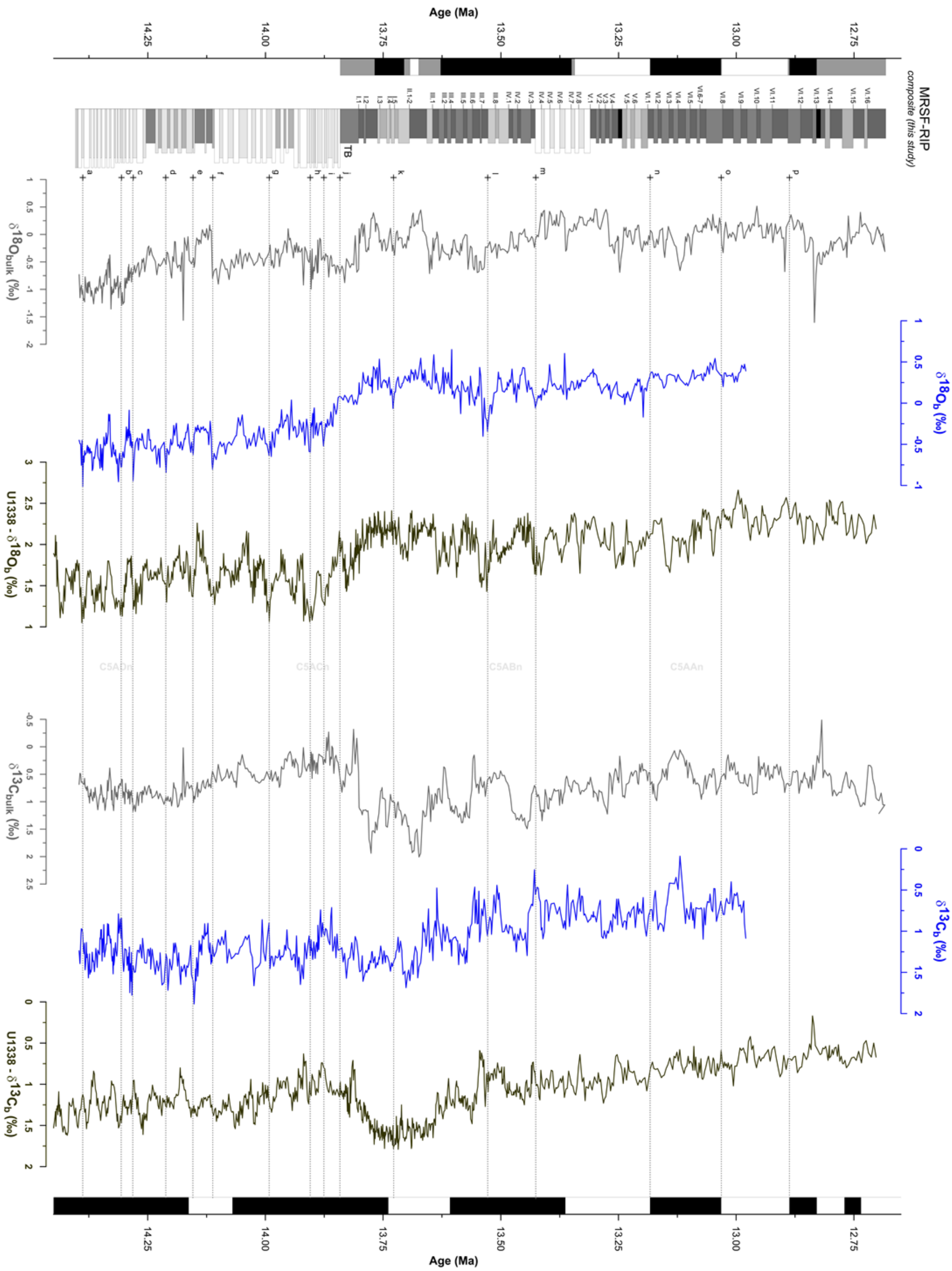

Figure S2: Carbonate stable isotope with age for Marsalforn and Ras il-Pellegrin (MRSF-RIP) sections.

Tie points used to produce the age model.

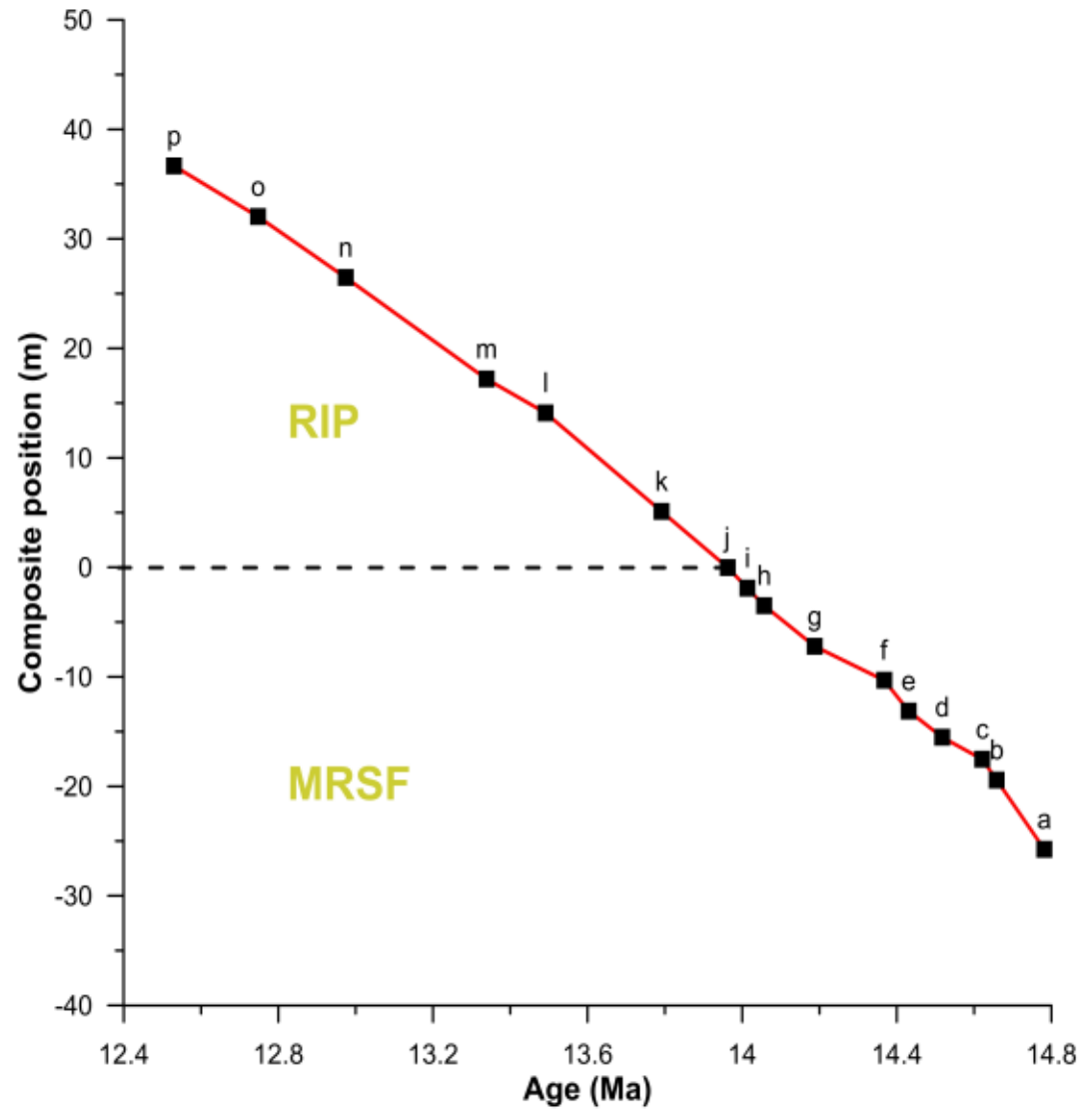

Figure S3: Depth vs age for MRSF-RIP. Age model for the composite section. MRSF-Marsalforn section, RIP-Ras il-Pellegrin section.

Section	Tie point	Age (Ma) Ceno GRID	height in section (m)	Magnetic polarity
MRSF	a	14.388	-25.75	
MRSF	b	14.306	-19.43	
MRSF	c	14.281	-17.53	
MRSF	d	14.212	-15.52	
MRSF	e	14.154	-13.12	
MRSF	f	14.112	-10.32	
MRSF	g	13.992	-7.21	
MRSF	h	13.905	-3.50	
MRSF	i	13.876	-1.90	
MRSF	j	13.842	-0.02	
RIP	k	13.728	5.10	
RIP	l	13.528	14.10	
RIP	m	13.426	17.20	
RIP	n	**13.183**	26.47	**C5Aan (average)**
RIP	o	**13.032**	32.03	**C5Ar.2r (average)**
RIP	p	**12.887**	36.66	**C5Ar.2n (average)**

Table S1: CenoGRID tie points for MRSF-RIP. Age model based on CenoGRID. MRSF ─ Marsalforn section, RIP – Ras il-Pellegrin section.

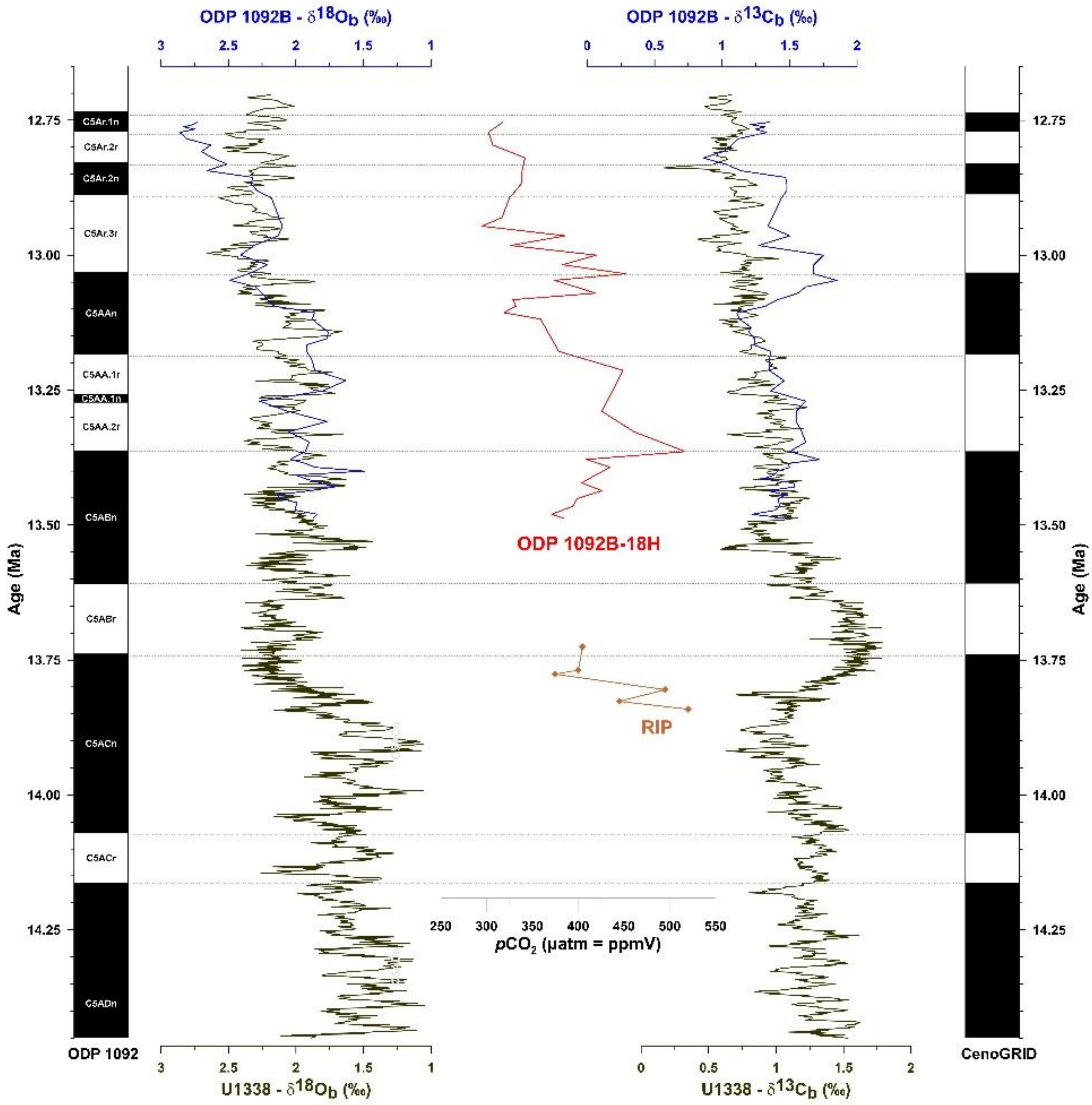

Figure S4:Magnetostratigraphic and carbonate stable isotope age for ODP 1092B, Southern Ocean. Magnetostratigraphy (*47*) used in conjunction with stable isotope data (*48*) and $\delta^{11}B$ based pCO_2 (*24*)from the same site in order tune these records to CenoGRID (*4*).

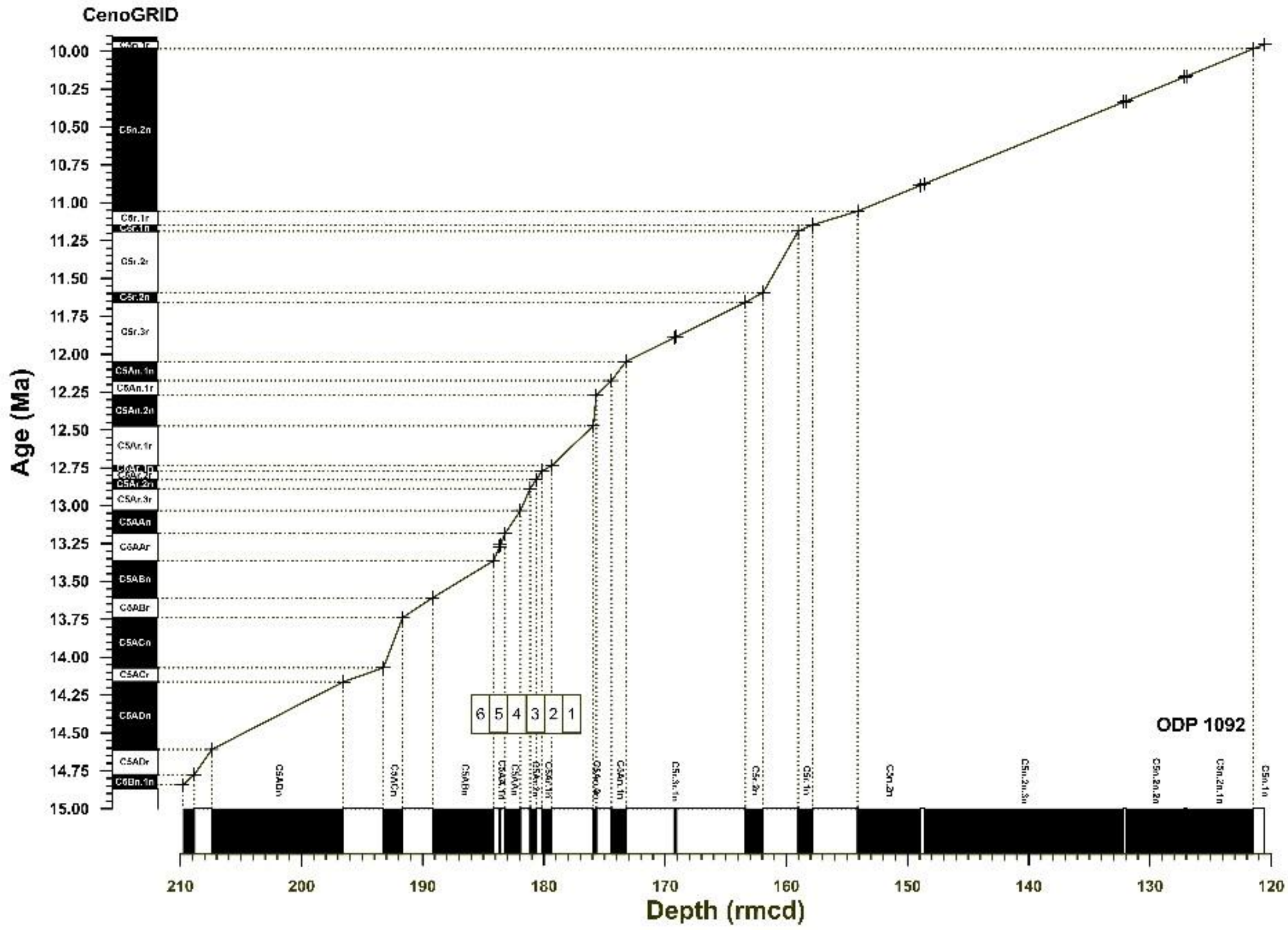

Figure S5 Age vs Depth ODP 1092B: Magnetostratigraphic tie points ODP 1092 (*47*)

Depth II (mcd)	Depth III (rmcd)	Offset III-II (m)	Reversal	Chron	Age (Ma) CK92	Age (Ma) Kuhnert (2009)	Age (Ma) Raitzsch (2021)	Age (Ma) Ceno GRID (This study)	Reference
121.48	120.55	-0.93	Base	C5n.1n	9.88				Evans et al (*46*)
122.4	121.47	-0.93	Top	C5n.2n	9.92				Evans et al (*46*)
127.92	127	-0.92	Top	C5n.2n.1	10.098*				Evans et al (*46*)
128.01	127.15	-0.86	Base	C5n.2n.1	10.103*				Evans et al (*46*)
132.88	131.95	-0.93	Top	C5n.2n.2	10.258*				Evans et al (*46*)
133.02	132.16	-0.86	Base	C5n.2n.2	10.263*				Evans et al (*46*)
151.31	148.6	-2.71	Top	C5n.2n.3	10.803*				Evans et al (*46*)
151.43	148.95	-2.48	Base	C5n.2n.3	10.814*				Evans et al (*46*)
156.6	154.12	-2.48	Base	C5n.2n	10.949				Evans et al (*46*)
158.8	157.82	-0.98	Top	C5r.1n	11.052				Evans et al (*46*)
160	159.02	-0.98	Base	C5r.1n	11.099				Evans et al (*46*)
163.1	161.91	-1.19	Top	C5r.2n	11.476				Evans et al (*46*)
164.54	163.37	-1.17	Base	C5r.2n	11.531				Evans et al (*46*)
170.6	169.07	-1.53	Top	C5r.3r.1n	11.866*				Evans et al (*46*)
170.79	169.26	-1.53	Base	C5r.3r.1n	11.877*				Evans et al (*46*)
171.75	173.24	1.49	Top	C5An.1n	11.935				Evans et al (*46*)
173	174.42	1.42	Base	C5An.1n	12.078				Evans et al (*46*)
174.2	175.67	1.47	Top	C5An.2n	12.184				Evans et al (*46*)
174.49	175.94	1.45	Base	C5An.2n	12.401				Evans et al (*46*)
**177.7**	**179.35**	**1.65**	**Top**	**C5Ar.1n**	**12.678**	**13.162**	**13.151**	**12.735**	Evans et al (*46*)
**178.5**	**180.15**	**1.65**	**Base**	**C5Ar.1n**	**12.708**	**13.230**	**13.219**	**12.770**	Evans et al (*46*)
**179**	**180.65**	**1.65**	**Top**	**C5Ar.2n**	**12.775**	**13.283**	**13.275**	**12.829**	Evans et al (*46*)
**179.42**	**181.13**	**1.71**	**Base**	**C5Ar.2n**	**12.819**	**13.420**	**13.422**	**12.887**	Evans et al (*46*)
**180.29**	**181.94**	**1.65**	**Top**	**C5AAn**	**12.991**	**13.710**	**13.730**	**13.032**	Evans et al (*46*)
**181.55**	**183.2**	**1.65**	**Base**	**C5AAn**	**13.139**	**14.090**	**13.957**	**13.183**	Evans et al (*46*)
**181.95**	**183.6**	**1.65**	**Top**	**C5AAr.1n**	**13.208***	**14.207**	**14.004**	**13.259**	Evans et al (*46*)
**182.02**	**183.67**	**1.65**	**Base**	**C5AAr.1n**	**13.220***	**14.215**	**14.011**	**13.272**	Evans et al (*46*)
**182.5**	**184.15**	**1.65**	**Top**	**C5ABn**	**13.302**	**14.262**	**14.077**	**13.363**	Evans et al (*46*)
187.47	189.19	1.72	*Base*	*C5ABn*				13.608	Evans and Channell (*47*)
192.43	191.66	-0.77	*Top*	*C5ACn*				13.739	Evans and Channell (*47*)
194.01	193.24	-0.77	*Base*	*C5ACn*				14.070	Evans and Channell (*47*)
197.32	196.55	-0.77	*Top*	*C5ADn*				14.163	Evans and Channell (*47*)
208.17	207.4	-0.77	*Base*	*C5ADn*				14.609	Evans and Channell (*47*)
209.61	208.84	-0.77	*Top*	*C5Bn.1n*				14.775	Evans and Channell (*47*)

Table S2: Middle Miocene Tie points for ODP1092. ODP 1092 age model aligned to CenoGRID. *From short polarity and not represented on GPTS. Rows in bold indicate tie points used in this present study. The ages of C5AAr.1n (top) and C5AAr.1n (base) are not given in CenoGRID, so these were interpolated ages.

Section 2 – Microfossil preservation and integrity of the geochemical records

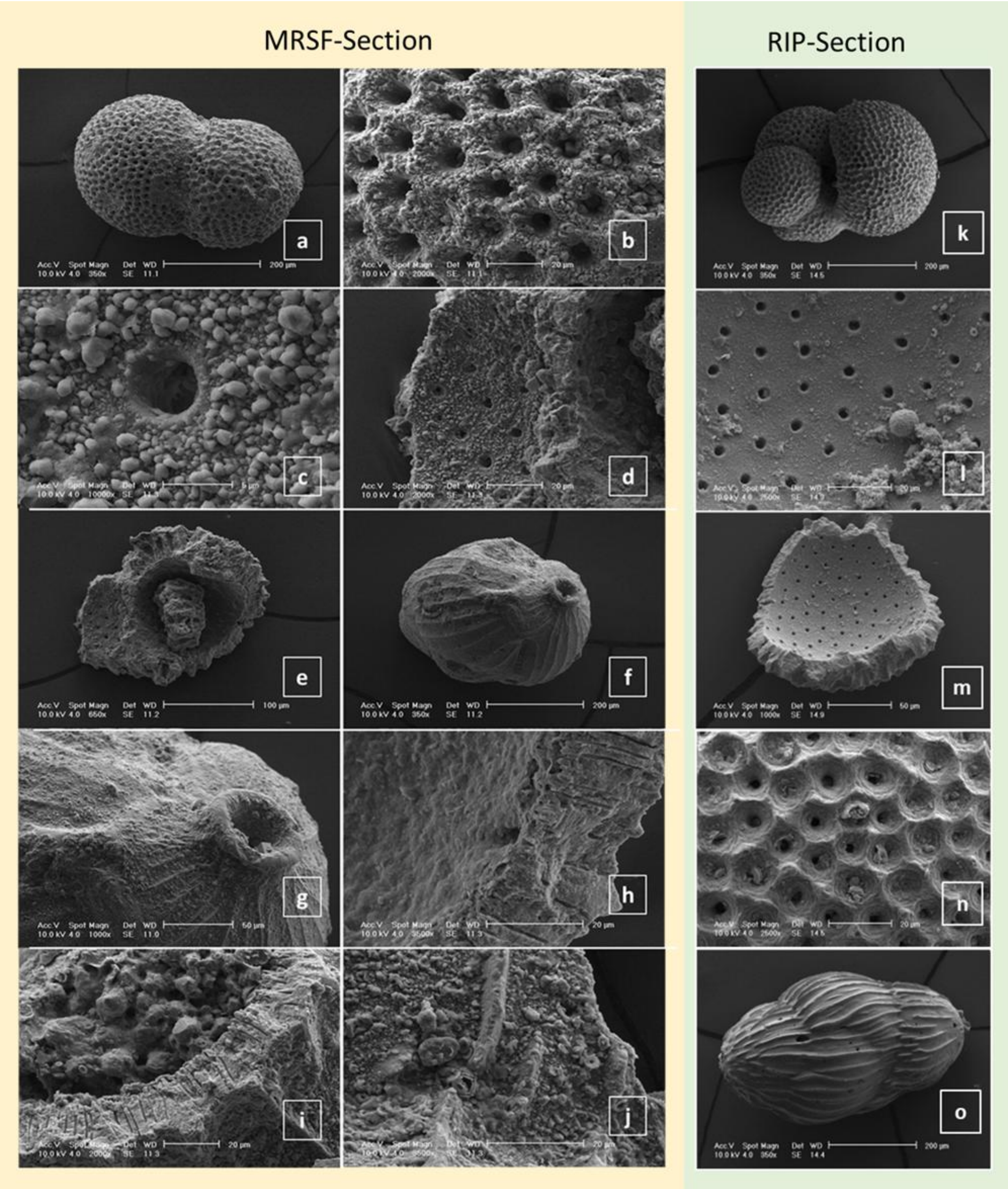

Figure S6: SEM images of MRSF-RIP microfossils: Planktic and benthic individual specimens from sample MT 1722 from the MRSF section compared (yellow panel) with planktic and benthic specimens from the RIP section (green panel). MRSF section (a)(b) original hexagonal pore space in *T. trilobus* compromised by recrystallisation of test calcite (c) authigenic crystals visible on inner test wall of *T. trilobus* (d)(e) pores through *T. trilobus* test wall compromised by diagenetic processes (f)(g) authigenic crystal growth on exterior surface of *Uvigerina* spp. including a single prominent calcite crystal adhering to the aperture (h)(i) presence of straight-edged pores through *Uvigerina* spp. test wall demonstrate lack of wholesale recrystallisation in this benthic foraminifer (j) coccolith cements and authigenic overgrowths on the interior surface of *Uvigerina* spp. RIP section (k)(l)(m)(n) whole and interior and exterior fragments from *T. trilobus* showing excellent preservation of original test material. (o) excellently preserved whole specimen of *Uvigerina* spp .

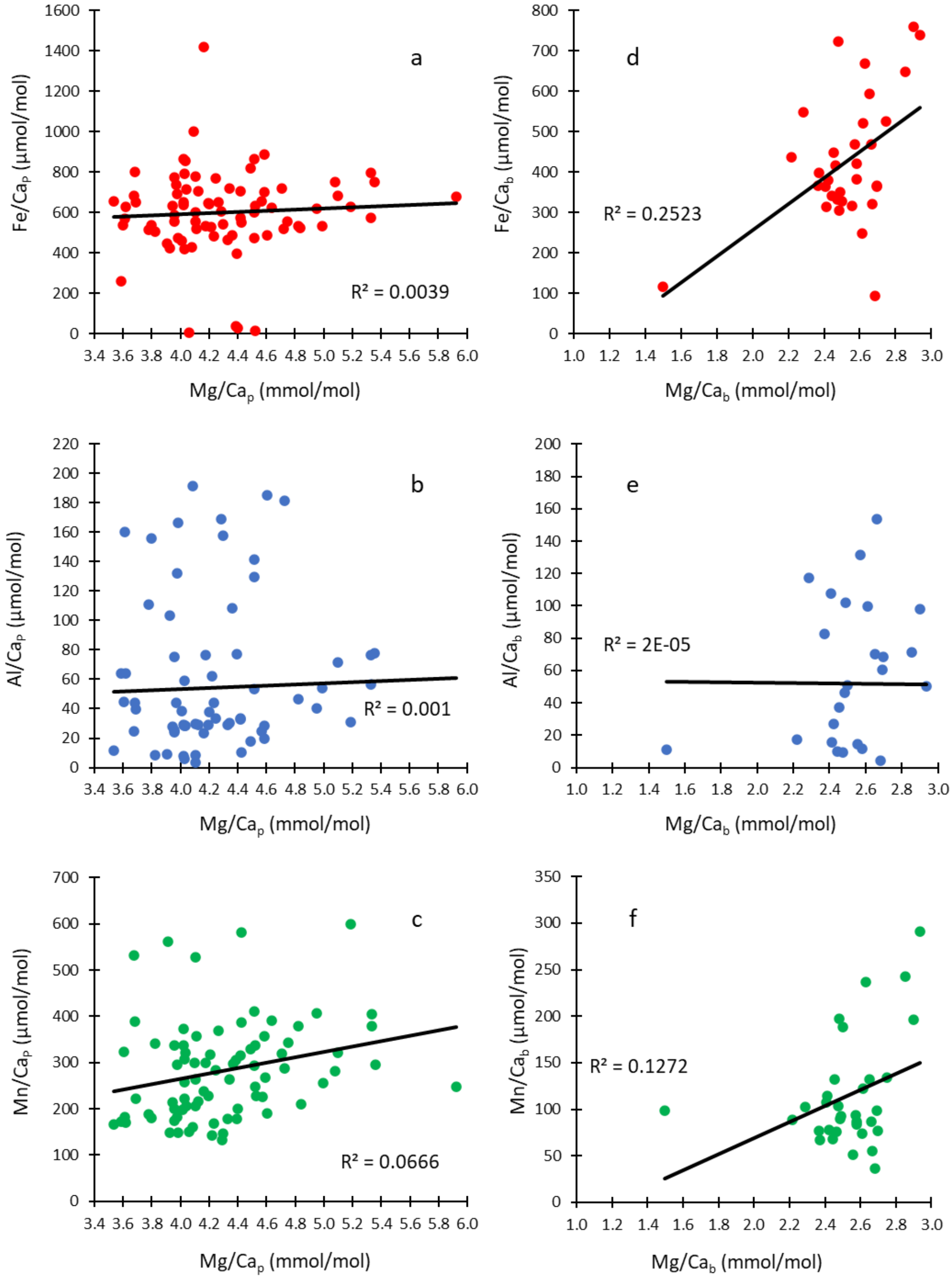

Figure S7: Assessment of contaminants in foraminiferal Mg/Ca. Correlations between possible contaminants for the Mg/Ca record from the RIP section. Fe/Ca, Al/Ca and Mn/Ca correlations with Mg/Ca (a)(b)(c) planktic record and (d)(e)(f) benthic record. No significant correlations are noted in either the planktic or the benthic record.

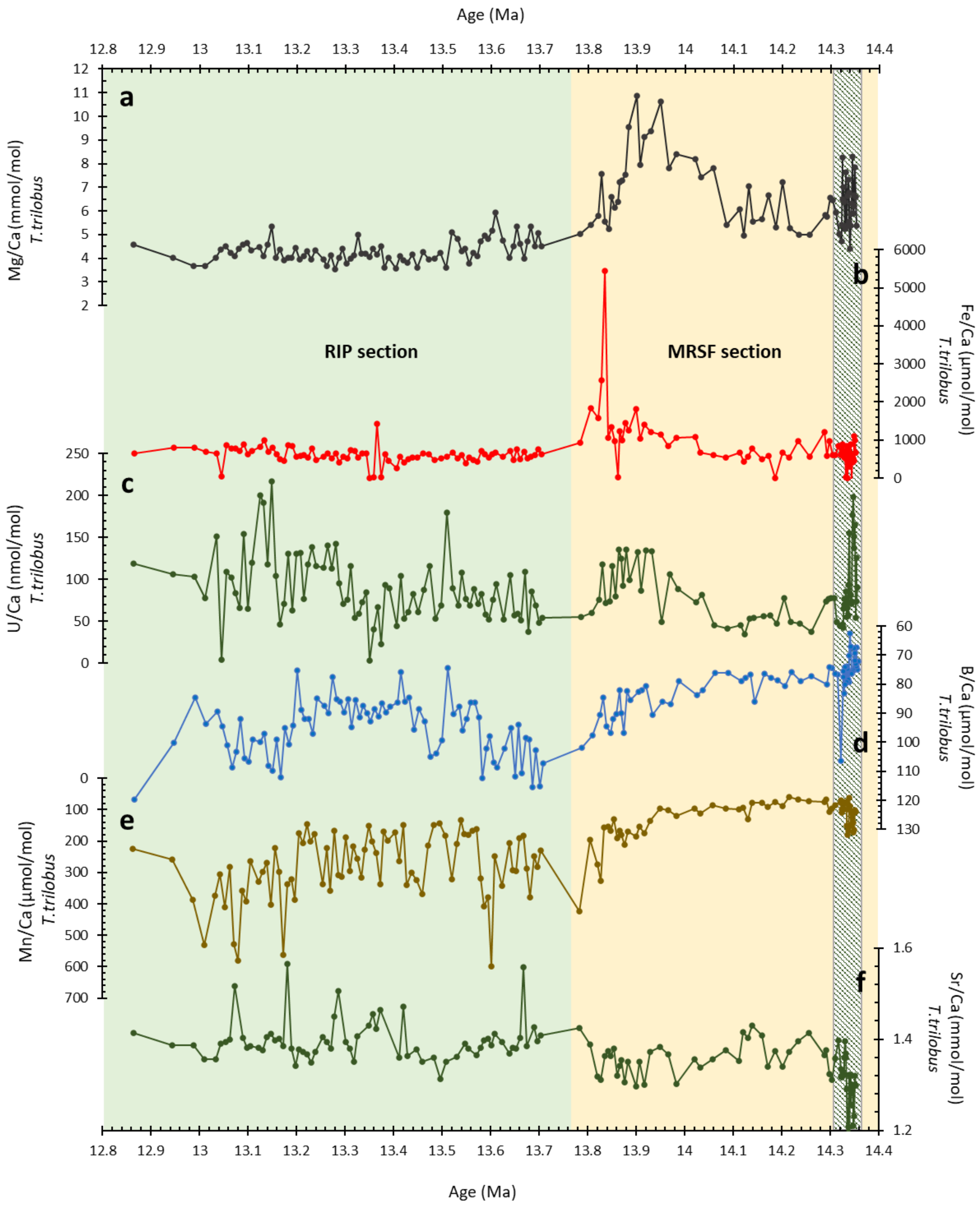

Figure S8: Foraminiferal Mg/Ca compared with X/Ca for identification of contaminant mineral phases. Comparison of *T. trilobus* (a) Mg/Ca, (b) Fe/Ca, (c) U/Ca, (d) B/Ca, (e) Mn/Ca and (f) Sr/Ca in order to asses preservation of original geochemical signal. (a)(b)(c) Close correlation between Mg/Ca, Fe/Ca and U/Ca for most of the MRSF section indicates that contaminant phases rich in U and Fe exclude the possibility of using the Mg/Ca from the MRSF section to obtain a palaeotemperature record. No correlation is observed between B/Ca (d) and U/Ca (c) and Fe/Ca (b). There is also no correlation between Mn/Ca (e) and U/Ca (c) and Fe/Ca (b). Sr/Ca (f) is relatively stable throughout the whole interval except for large excursions in the oldest part of the record. Grey hatched bar indicates part of record likely impacted by recrystallisation.

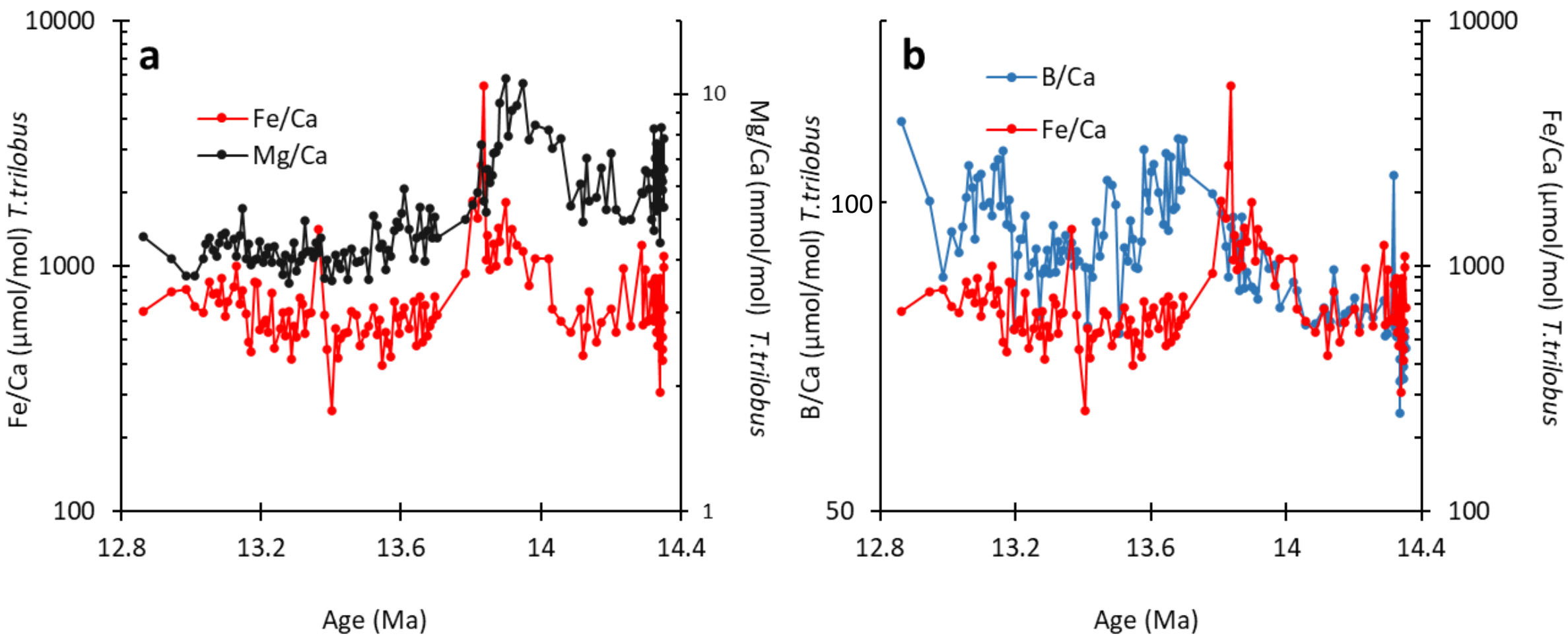

Figure S9: Assessment of Fe-related contamination. (a) Clear association between planktic foraminiferal Fe/Ca and Mg/Ca in the older (14.4 Ma to 13.8 Ma) part of the record indicating a contaminant phase containing both Fe and Mg, likely an Fe-dolomite. (b) There is no clear co-variation between planktic Fe/Ca and B/Ca, suggesting that the B/Ca record is not impacted by the presence of Fe-dolomite.

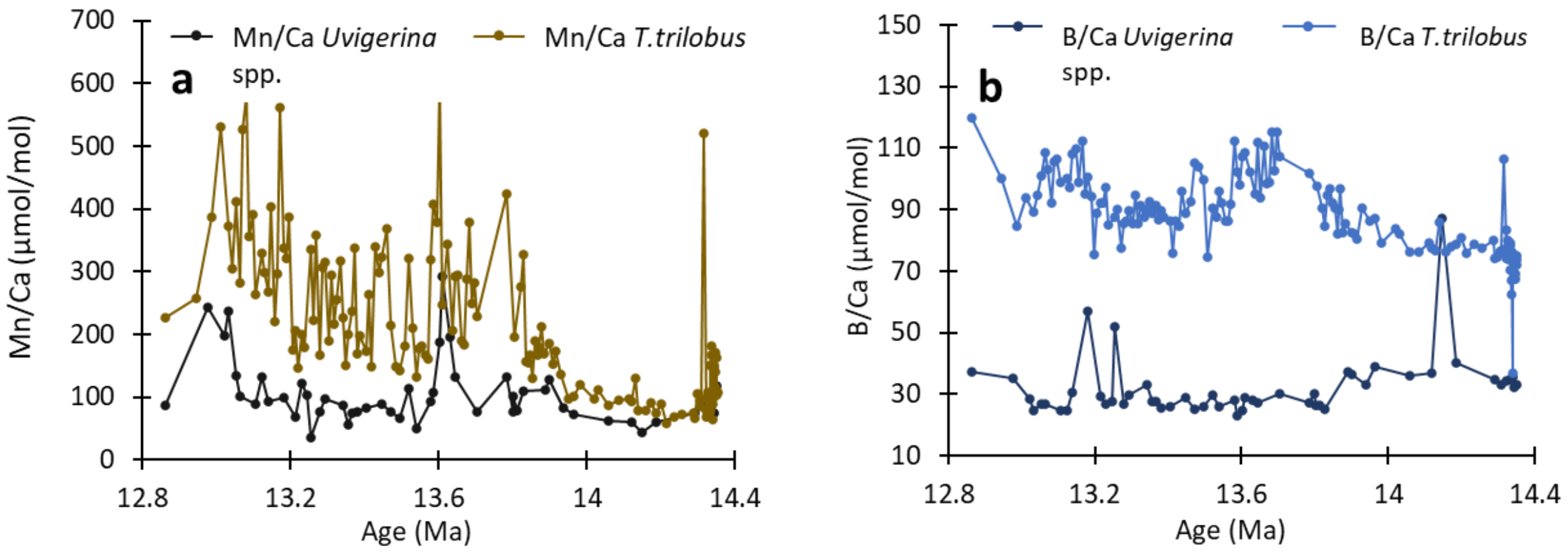

Figure S10: Comparison of B/Ca and Mn/Ca planktic and benthic records. (a) Planktic and benthic Mn/Ca records show close covariation for most of the studied interval (b) no identifiable covariation is observed in the B/Ca benthic and planktic records.

Section 3 –Anova single factor analysis $\delta^{18}O$ and $\delta^{13}C$ MRSF-RIP records

Groups	*Count*	*Sum*	*Average*	*Variance*
$\delta^{13}C_p$ ‰(VPDB) (Mourik et al (*9*)) pre Mi-3b	362	729.94	2.01641	0.06593
$\delta^{18}O_p$ ‰(VPDB) (Mourik et al (*9*))pre Mi-3b	362	-574.15	-1.58605	0.03819
$\delta^{13}C_b$ ‰(VPDB) (Mourik et al (*9*))pre Mi-3b	369	458.95	1.24377	0.03929
$\delta^{18}O_b$ ‰(VPDB) (Mourik et al (*9*))pre Mi-3b	367	-142.47	-0.38820	0.04402
$\delta^{13}C_p$ ‰(VPDB) (Mourik et al (*9*)) post Mi-3b	334	734.75	2.19985	0.17503
$\delta^{18}O_p$ ‰(VPDB) (Mourik et al (*9*)) post Mi-3b	334	-511.81	-1.53237	0.07380
$\delta^{13}C_b$ ‰(VPDB) (Mourik et al (*9*)) post Mi-3b	327	314.15	0.96070	0.09088
$\delta^{18}O_b$ ‰(VPDB) (Mourik et al (*9*)) post Mi-3b	326	75.43	0.23138	0.01954

Table S3: Average and variance analysis for MRSF-RIP benthic and planktic $\delta^{18}O$ and $\delta^{13}C$ records. Anova single factor for benthic and planktic stable isotope records $\delta^{18}O$ and $\delta^{13}C$.
